# Donepezil for Fatigue and Psychological Symptoms in Post–COVID-19 Condition: A Randomized Clinical Trial

**DOI:** 10.1001/jamanetworkopen.2025.0728

**Published:** 2025-03-17

**Authors:** Kensuke Nakamura, Kazuhiro Kondo, Naomi Oka, Kazuma Yamakawa, Kenya Ie, Tadahiro Goto, Shigeki Fujitani

**Affiliations:** 1 Department of Critical Care Medicine, Yokohama City University Hospital, Yokohama, Japan; 2 Department of Fatigue Science, Jikei University School of Medicine, Tokyo, Japan; 3 Department of Virology, Jikei University School of Medicine, Tokyo, Japan; 4 Department of Emergency and Critical Care Medicine, Osaka Medical and Pharmaceutical University, Takatsuki, Japan; 5 Department of General Medicine, Kawasaki Municipal Tama Hospital, Kawasaki, Japan; 6 TXP Medical Co Ltd, Tokyo, Japan; 7 Department of Emergency and Critical Care Medicine, St Marianna University School of Medicine, Kawasaki, Japan

## Abstract

**Importance:**

Fatigue is the most commonly reported symptom of post–COVID-19 condition (also known as long COVID) and impairs various functions. One of the underlying mechanisms may be intracerebral inflammation due to decreases in acetylcholine levels.

**Objective:**

To examine the effects of donepezil hydrochloride, an acetylcholinesterase inhibitor, on post–COVID-19 fatigue and psychological symptoms.

**Design, Setting, and Participants:**

A multicenter, double-blind randomized clinical trial was performed in Japan. Between December 14, 2022, and March 31, 2024, adult patients within 52 weeks of the onset of COVID-19 and with a global binary fatigue score of 4 or greater on the Chalder Fatigue Scale were randomized into a donepezil or a placebo group.

**Exposure:**

The intervention was conducted during a 3-week period, with donepezil hydrochloride being administered at a dosage of 3 mg/d for the first week and then 5 mg/d for 2 weeks.

**Main Outcomes and Measures:**

The primary outcome was a change in the Chalder Fatigue Scale score and the absolute score 3 weeks after the initiation of treatment. Other outcomes at 3 and 8 weeks, such as psychological symptoms and quality of life, were evaluated as secondary outcomes.

**Results:**

A total of 120 eligible patients were enrolled and 10 withdrew or were lost to follow-up; therefore, 110 patients (55 in each group) were included in the efficacy analysis (64 [58%] female; mean [SD] age, 43 [12] years). No significant differences were observed in baseline characteristics between the 2 groups. The baseline-adjusted estimating treatment effect of donepezil, measured as the mean difference on Chalder Fatigue Scale scores at 3 weeks, was 0.34 (95% CI, −2.23 to 2.91), showing no significant effect of the intervention (*P* = .79). Scores for the Hospital Anxiety and Depression Scale, Impact of Event Scale–Revised, EuroQol 5-Dimension 5-Level Version, Patient Health Questionnaire, and Daily Health Status at 3 and 8 weeks were similar. No serious adverse events occurred in either group.

**Conclusions and Relevance:**

In this randomized clinical trial of donepezil to treat post–COVID-19 condition, the efficacy for fatigue and psychological symptoms was not confirmed in a general population. The development of effective therapeutics for post–COVID-19 symptoms is needed, and more clinical trials should be conducted in the future.

**Trial Registration:**

Japan Registry of Clinical Trials Identifier: jRCT 2031220510

## Key Points

**Question:**

Can fatigue and psychological symptoms in post–COVID-19 condition be alleviated with donepezil hydrochloride, an acetylcholinesterase inhibitor?

**Findings:**

In this randomized clinical trial of 110 patients, the baseline-adjusted estimating treatment effect of donepezil on Chalder Fatigue Scale scores at 3 weeks was an increase of 0.34, which was not statistically significant. Secondary outcomes including depression, anxiety, and quality of life scores at 3 and 8 weeks were also negative.

**Meaning:**

These findings suggest that donepezil should not be used for fatigue and psychological symptoms in post–COVID-19 condition in a general population.

## Teaser

This randomized clinical trial investigates whether donepezil may be effective against post–COVID-19 fatigue and psychological symptoms among patients within 1 year of COVID-19 diagnosis.

## Introduction

The pandemic phase of COVID-19 has ended; however, new cases are still emerging worldwide.^1^ Symptoms may persist even after recovery from acute COVID-19,^2^ and long-lasting sequelae have become a social issue, termed post–COVID-19 condition (also known as long COVID).^3,4^ Fatigue is the most commonly reported symptom, affecting 40% to 60% of patients,^5,6,7^ and impairments in various functions make it difficult to reintegrate into society.^8^ Moreover, psychiatric symptoms, such as anxiety, depression, and posttraumatic stress disorder, are common in post–COVID-19 condition and associated with fatigue.^9,10^ These symptoms currently affect more than 60 million individuals worldwide; however, this number is increasing.^11^

Although the development of treatments for post–COVID-19 condition is urgently needed, there is still no evidence-based recommended medication.^12^ Since the underlying mechanisms remain unclear, difficulties are associated with selecting appropriate therapeutic approaches.^13^ We previously reported that decreases in both the number of intracerebral cells producing acetylcholine and acetylcholine concentrations were associated with brain inflammation and the clinical symptoms of fatigue and/or depression in a mouse model of COVID-19 in which the S1 protein, a component of the COVID-19 virus, was intranasally administered.^14^ Donepezil hydrochloride, a cholinesterase inhibitor, has been shown to attenuate brain inflammation and improve clinical symptoms in this model, even with a converted dose used for dementia.^14^ The mechanisms responsible for fatigue and/or depression may involve one of the human herpesvirus 6 (HHV-6) proteins, SITH-1, a small protein encoded by the intermediate-stage transcript of HHV-6^15^; therefore, HHV-6 infection or reactivation has been suggested to induce fatigue^16^ and depression.^17^ Since COVID-19 infection is more likely to be accompanied by herpesvirus reactivation,^18^ with HHV-6 being the most frequently involved,^19^ donepezil may be effective for these patients. We herein hypothesized that donepezil may be effective against post–COVID-19 fatigue and psychological symptoms and conducted a randomized clinical trial (RCT) among patients after COVID-19 infection.

## Methods

### Design

A multicenter, placebo-controlled, double-blind, superiority, 1:1 allocated phase 2 RCT was performed in Japan to investigate the efficacy of donepezil for mild to moderate COVID-19 with fatigue symptoms. The study protocol was approved by the Ethics Committee of St Marianna Medical University Hospital. The trial protocol was described in detail in a previous protocol report^20^ and is found in Supplement 1. All participants provided written informed consent or e-consent before enrollment. This report follows the Consolidated Standards of Reporting Trials (CONSORT) reporting guidelines for RCTs.

### Participants and Settings

Between December 14, 2022, and March 31, 2024, consecutive patients with fatigue symptoms after COVID-19 infection were enrolled. Because scheduled enrollment was achieved early, patient registration was stopped and the study was completed on December 31, 2023.

Inclusion criteria were as follows: (1) age between 20 and 75 years at the time of consent; (2) COVID-19 infection and an upper respiratory tract infection, fever, or cough in the acute phase; (3) a global binary fatigue score of 4 or greater on the Chalder Fatigue Scale (CFS) before the intervention; (4) a positive COVID-19 antigen or polymerase chain reaction test result within 21 days from the onset of COVID-19 to randomization; and (5) provision of consent themselves. The inclusion criteria were revised in February 2023. Since more than half of patients with post–COVID-19 condition tested positive for the SITH-1 antibody within 1 year (positive rate of 7.3% in healthy individuals) in another ongoing study (N.O., K.N., Koichi Hirahata, MD, et al; unpublished data), criterion 4 was revised to expand the period to within 52 weeks to increase the number of eligible patients. Similarly, part of criterion 2, “those who did not have a fever higher than 38.0°C for 24 hours,” was deleted. Exclusion criteria were as follows: patients with a previous diagnosis or suspected diagnosis of psychiatric disorders or chronic fatigue syndrome relevant to codes F0 to F3 in the *International Statistical Classification of Diseases, Tenth Revision*; respiratory disease (Hugh-Jones class ≥2); heart failure (New York Heart Association class ≥2); undergoing dialysis or likelihood of starting dialysis; cirrhosis (Child-Pugh class C); pregnancy; influenza infection; current use of donepezil or anticholinesterase inhibitors; inability to answer the questionnaire in person (including those with cognitive decline); an allergy to any component of the investigational drug; a history of hypersensitivity to piperidine derivatives; and previous participation in this study. Treatment introduced in the acute phase of COVID-19 was not relevant to inclusion.

### Interventions

Patients were randomized in a 1:1 ratio to the donepezil or the placebo group. This trial was performed in a double-blind manner until its completion. Among participants in isolation, treatments and follow-ups were conducted via telemedicine or home visits. In the donepezil group, a low dosage of 3 mg/d was administered for 1 week. If no adverse events were observed, the dosage was increased to 5 mg/d for 2 weeks. This is the default dosage for dementia in Japan, and sufficient efficacy was confirmed in the results of the basic studies by Oka et al.^14^ In the control group, patients took a dosage of lactose, 0.6 g/d for 1 week, followed by 1 g/d for 2 weeks as the placebo. The concomitant use of antiepileptic drugs, antipsychotics, antidepressants, anxiolytics, central stimulants, antiparkinsonian drugs, and antipyretics was prohibited. Prohibited therapies included repetitive transcranial magnetic stimulation therapy and epipharyngeal abrasive therapy.

### Outcomes

Outcome symptom surveys were evaluated based on the CFS, the Hospital Anxiety and Depression Scale (HADS), Impact of Event Scale–Revised (IES-R), EuroQol 5-Dimension 5-Level Version (EQ-5D-5L), Patient Health Questionnaire (PHQ-9), and Daily Health Status (DHS). The investigation on outcome symptoms conducted online 3 and 8 weeks after the start of treatment used questionnaires. The intervention was discontinued at 3 weeks; however, we also followed up patients for 8 weeks to identify whether post–COVID-19 conditions were inhibited in that period. The primary end point was a change in CFS score and the absolute CFS value^21^ at 3 weeks. Secondary end points were a change in CFS score and the absolute CFS score at 8 weeks and scores on the HADS, IES-R, EQ-5D-5L, PHQ-9 and DHS at 3 and 8 weeks. The medication adherence rate was assessed based on unused medication and empty bags, and less than 80% was counted as a deviation.

### Adverse Events Reporting, Monitoring, and Interim Analysis

Adverse events were collected 1, 3, and 8 weeks after administration, and those attributed to the study drug were examined. Severe adverse events were to be immediately reported to the principal investigator (K.N.), who would report them to each investigator within a specific number of days according to their severity. The principal investigator had the authority to terminate the study if a marked difference was noted in safety based on reports of severe adverse events or safety monitoring.

The monitoring manager (S.F.) confirmed that the human rights and safety of participants were protected and that the study was being conducted in accordance with the latest study protocol by checking against source documents and records. Auditing in the present study was conducted by Multiplex Limited Liability Company. Protocol adherence and input data were checked by the monitoring committee. Interim analyses were not performed.

### Sample Size Estimation

Based on previous studies that examined the CFS in patients with COVID-19,^22,23,24^ we set the mean (SD) CFS score to 15 (5) (range, 0-33, with higher scores indicating greater fatigue severity) and the expected minimal important difference to a decrease of 3 points.^25^ With an α error of .05 and β error of 0.20, each group required 45 patients, for a total of 90 patients. Accounting for an anticipated discontinuation rate of 25%, the final sample size was set at 120 participants (60 in each group). According to revisions to the inclusion criteria, we referenced another study in which the patient population was similar.^26,27,28^ We estimated mean (SD) CFS scores of 15 (5) at 1 year after the onset of COVID-19 and set the minimal important difference to a 3-point decrease in the CFS score. Given an α error of .05 and β error of 0.20, each group required 45 patients, resulting in 90 patients. In addition, even when accounting for an anticipated discontinuation rate of 25%, a sample size of 120 participants (60 in each group) was considered to be sufficient. Further details are described in the protocol.^20^

### Statistical Analysis

Statistical analyses were performed with a full analysis set (FAS), a safety analysis set (SAS), and a per-protocol set (PPS). The FAS was defined as all participants without violations in the main eligibility criteria (selection and exclusion criteria) or conflicts with discontinuation and dropout criteria. The SAS was defined as all participants who received the study treatment. If the adherence rate was less than 80% at the end of the trial or at the time of discontinuation, it was treated as an exclusion in the PPS. An efficacy analysis was performed with the FAS. Adverse events were analyzed with SAS. A sensitivity analysis of efficacy was performed with the PPS. Participants with missing data on the primary or secondary outcomes were not included in the FAS and PPS to evaluate outcomes. Other missing data were treated as missing values with a missing indicator.

Outcomes were compared as a change in each score by unadjusted analysis and by regression models adjusted for baseline CFS scores and for baseline CFS scores plus age, sex, disease severity, medical history (endocrine, digestive, allergy, mental, neurological, respiratory, and cardiovascular), and cotreatment (acupuncture therapy) (analysis of covariance) and as the absolute value by unpaired *t* test. Since there were 3 missing values at 8 weeks, we used case complete analysis. Subgroup analyses were performed based on age, sex, medical history, and disease severity, measured as more than 1 moderate symptom by self-report in the first week of COVID-19 infection or not.^29^ Analyses were performed using R, version 4.4.1 (R Project for Statistical Computing). Two-sided *P* < .05 was considered statistically significant.

## Results

**Figure 1.**
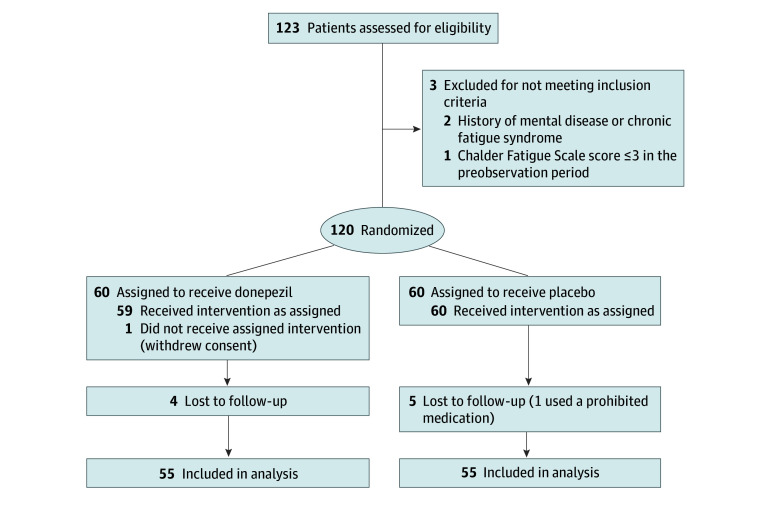
Participant Flow Diagram

A total of 120 eligible patients were enrolled; 60 were randomized to the donepezil group and 60 to the placebo group. The participant flow diagram is shown in Figure 1. Since 1 patient withdrew consent, 119 were assigned to the SAS for the assessment of adverse events; 4 patients in each group dropped out before the outcome assessment at 3 weeks; and 1 patient was later found to be concomitantly using a prohibited medication before the initiation of the study and stopped the follow-up. Thus, 110 patients (55 in each group) were assigned to the FAS to assess outcomes (64 [58%] female and 46 [42%] male; mean [SD] age, 43 [12] years). Three-week mean (SD) adherence rates in the FAS were 99.4% (0.4%) in the donepezil group and 99.0% (0.4%) in the control group (*P* = .70). One patient in each group had less than 80% medication adherence. In the control group, 1 patient was evaluated for the primary outcome outside of the allowance of days 19 to 21 from the start of medication, and 1 patient used a prohibited concomitant medication during the observation period. With the exclusion of these patients, 54 patients in the donepezil group and 52 in the control group were assigned to the PPS and included in the sensitivity analysis.

Table 1 shows basic characteristics in the FAS. Mean (SD) age was 45 (13) years in the donepezil group; 29 patients (53%) were female and 26 (47%) were male. Mean (SD) age in the control group was 40 (10) years; 35 patients (64%) were female and 20 (36%) were male. No abnormalities in vital signs or laboratory findings were noted in most patients. Furthermore, there were no obvious differences between previous histories and complications (eTable 1 in Supplement 2). eTable 2 in Supplement 2 shows the number of missing data for the observation items and outcomes other than the primary outcome. Missing outcomes 8 weeks after the intervention were also negligible in this study. eTable 3 in Supplement 2 shows basic characteristics in the patients lost to follow-up.

**Table 1.** Basic Patient Characteristics

Characteristic	Treatment group	
	Donepezil (n = 55)	Control (n = 55)
Age, mean (SD), y	45 (13)	40 (10)
Sex, No. (%)		
Female	29 (53)	35 (64)
Male	26 (47)	20 (36)
Time from symptom onset to participation		
Mean (SD), d	184 (127)	180 (123)
Range, d		
≤30	9 (16)	10 (18)
31-90	12 (22)	9 (16)
≥91	34 (62)	36 (65)
Concomitant therapy, No. of patients		
Acupuncture	6	17
Chiropractic or relaxation	2	3
CPAP	1	3
Elastic stockings	1	0
Glucose-ozone infusion	1	0
Hydrogen inhalation	1	0
Nicotinamide mononucleotide infusion	1	0
Symptoms on inclusion, No. (%)		
Cough	19 (35)	23 (42)
Sputum	21 (38)	14 (25)
Fever	7 (13)	3 (5)
Taste disorder	14 (25)	6 (11)
Appetite loss	18 (33)	20 (36)
No. of vaccinations, No. (%)		
0	5 (9)	10 (18)
1	0	2 (4)
2	9 (16)	8 (15)
3	22 (40)	19 (35)
4	10 (18)	13 (24)
5	8 (15)	3 (5)
6	1 (2)	0
Vital signs on inclusion, mean (SD)		
Body temperature, °C	36.6 (0.3)	36.7 (0.3)
Systolic blood pressure, mm Hg	121 (14)	117 (15)
Diastolic blood pressure, mm Hg	78 (11)	76 (12)
Heart rate, beats/min	73 (13)	75 (10)
Respiratory rate, breaths/min	15 (3)	15 (3)
Oxygen saturation, %	98 (1)	98 (1)
Laboratory findings on inclusion, mean (SD)		
White blood cells, No./μL	6090 (1960)	6190 (1560)
Neutrophils, %	59 (10)	60 (9)
Lymphocytes, %	31 (9)	31 (9)
Hemoglobin, g/dL	14.00 (1.43)	13.88 (1.33)
Platelets, No./μL	258 (67)	278 (66)
Albumin, g/dL	4.40 (0.39)	4.48 (0.25)
Aspartate aminotransferase, U/L	23 (8)	22 (8)
Alanine aminotransferase, U/L	26 (17)	25 (20)
Lactate dehydrogenase, U/L	170 (33)	176 (56)
Serum urea nitrogen, mg/dL	13.5 (6.6)	13.0 (3.8)
Creatinine, mg/dL	0.72 (0.24)	0.72 (0.15)
Hemoglobin A_1c_, %	5.44 (0.50)	5.38 (0.39)
C-reactive protein, mg/dL	0.15 (0.37)	0.16 (0.26)

Abbreviation: CPAP, continuous positive airway pressure.

SI conversion factors: To convert alanine aminotransferase to μkat/L, multiply by 0.0167; albumin to g/L, multiply by 10; aspartate aminotransferase to μkat/L, multiply by 0.0167; C-reactive protein to mg/L, multiply by 10; creatinine to μmol/L, multiply by 88.4; hemoglobin to g/L, multiply by 10; lactate dehydrogenase to μkat/L, multiply by 0.0167; platelets to ×10^9^/L, multiply by 0.001; serum urea nitrogen to mmol/L, multiply by 0.357; white blood cells to ×10^9^/L, multiply by 0.001.

**Figure 2.**
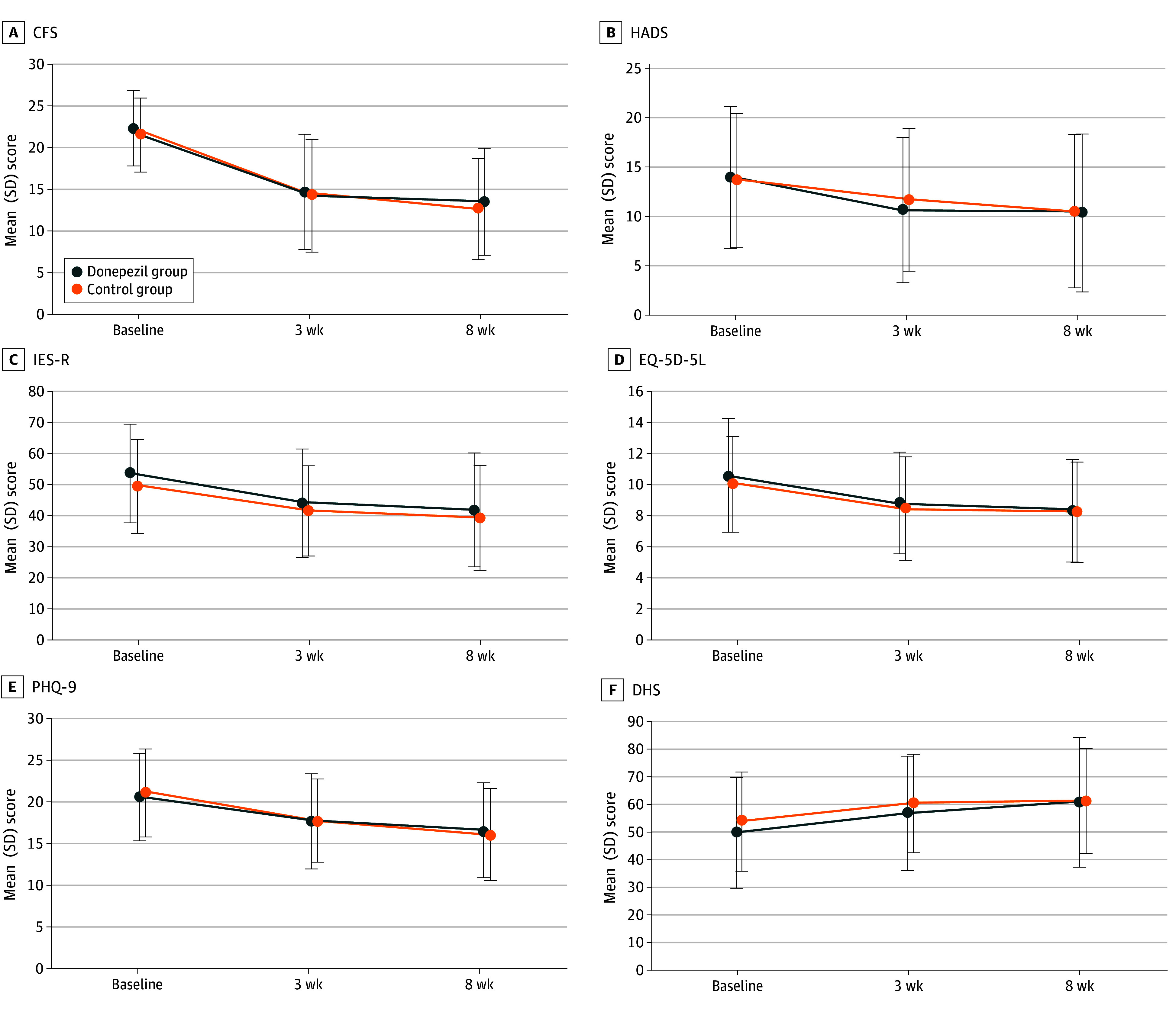
Outcome Trajectories Error bars indicate SDs. CFS indicates Chalder Fatigue Scale (scores range from 0-33, with higher scores indicating greater fatigue severity); DHS, Daily Health Status (scores range from 0-100, with higher scores indicating better health status); EQ-5D-5L, EurQol 5-Dimension 5-Level Version (scores range from 0-20, with higher scores indicating better quality of life); HADS, Hospital Anxiety and Depression Scale (scores range from 0-42, with higher scores indicating greater anxiety or depression severity); IES-R, Impact of Event Scale–Revised (scores range from 0-88, with higher scores indicating greater severity of posttraumatic stress disorder); and PHQ-9, Patient Health Questionnaire (scores range from 0-27, with higher scores indicating greater depression severity).

Regarding outcomes, the estimating treatment effect was adjusted for baseline points; evaluated changes in the scores from baseline are shown in Table 2 as the mean difference (95% CI) and *P* value. The absolute value of each outcome with *P* values is shown in eTable 4 in Supplement 2, and trajectories in the scores of each assessment are shown in Figure 2. No significant differences were observed between the 2 groups at each time point. The primary outcome, mean (SD) CFS at 3 weeks, was 14.6 (6.9) in the donepezil group and 14.1 (6.7) in the control group (*P* = .31) as the absolute value, and the estimating treatment effect adjusted for baseline CFS using a regression model was 0.34 (95% CI, −2.23 to 2.91), showing no significant effect of the intervention (*P* = .79). The estimating treatment effects unadjusted and adjusted for baseline CFS and prespecified confounders were similar. There was also no significant effect on the secondary outcomes, including CFS at 8 weeks or HADS, IES-R, EQ-5D-5L, PHQ-9, and DHS at 3 and 8 weeks. Similar results were observed in the sensitivity analysis in the PPS (eTable 5 in Supplement 2).

**Table 2.** Outcome Analysis of Donepezil Efficacy

Outcome	Estimating treatment effect, mean difference (95% CI)	*P* value
Primary (CFS at 3 wk)		
Unadjusted analysis	0.56 (−2.02 to 3.15)	.67
Adjusted for baseline CFS using regression model	0.34 (−2.23 to 2.91)	.79
Adjusted for baseline CFS and prespecified confounders using regression model^a^	−0.40 (−3.15 to 2.34)	.77
Secondary		
CFS at 8 wk	−1.06 (−3.38 to 1.26)	.37
HADS at 3 wk	−1.34 (−3.20 to 0.53)	.16
HADS at 8 wk	−0.07 (−2.47 to 2.33)	.95
IES-R at 3 wk	−0.73 (−5.07 to 3.61)	.74
IES-R at 8 wk	−0.08 (−5.86 to 5.71)	.98
EQ-5D-5L at 3 wk	0.01 (−0.88 to 0.89)	.99
EQ-5D-5L at 8 wk	−0.29 (−1.23 to 0.62)	.52
PHQ-9 at 3 wk	0.26 (−1.23 to 1.75)	.73
PHQ-9 at 8 wk	0.69 (−1.33 to 2.70)	.50
Daily Health Status at 3 wk	−1.76 (−8.30 to 4.79)	.60
Daily Health Status at 8 wk	0.74 (−6.75 to 8.23)	.85

Abbreviations: CFS, Chalder Fatigue Scale; HADS, Hospital Anxiety and Depression Scale; IES-R, Impact of Event Scale–Revised; EQ-5D-5L, EuroQol 5-Dimension 5-Level Version; PHQ-9, Patient Health Questionnaire.

^a^ Adjusted for age, sex, disease severity, medical history (endocrine, digestive, allergy, mental, neurological, respiratory, and cardiovascular), and cotreatment (acupuncture therapy).

Adverse events are shown in Table 3. Twenty-one patients (36%) in the donepezil group and 25 (42%) in the control group had 1 or more adverse events. No serious adverse events occurred in either group. There were 12 adverse events (20%) in the donepezil group and 11 (18%) in the control group that were suspected to be causally related to the intervention, including central nervous system and gastrointestinal tract symptoms.

**Table 3.** Adverse Events

Event	Treatment group, No. (%) of patients	
	Donepezil (n = 59)	Control (n = 60)
≥1	21 (36)	25 (42)
Severe	0	0
With a causal relationship	12 (20)	11 (18)
Diarrhea	5 (8)	4 (7)
Headache	4 (7)	1 (2)
Nausea	3 (5)	0
Arousal during sleep	2 (3)	0
Stomachache	2 (3)	1 (2)
Fatigue	1 (2)	0
Cough	1 (2)	0
Nightmares	1 (2)	0
Sleepiness	1 (2)	0
Sleep disorder	1 (2)	1 (2)
Stomach discomfort	1 (2)	0
Abdominal distension	1 (2)	0
Prolonged COVID-19 symptoms	1 (2)	0
Depression	0	1 (2)
Fever	0	1 (2)
Heartburn	0	1 (2)
Suicidal thoughts	0	1 (2)
Postural tachycardia syndrome	0	1 (2)
Appetite loss	0	1 (2)

Subgroup analyses based on age, sex, medical history, and disease severity are shown in eTable 6 in Supplement 2. There was also no significant difference between the 2 groups.

## Discussion

We conducted an RCT to compare the effects of donepezil with a placebo for post–COVID-19 fatigue and psychological symptoms. Donepezil did not significantly affect the primary outcome of CFS score at 3 weeks or the secondary outcomes of fatigue, mental impairment, and quality of life measures at 3 and 8 weeks. Furthermore, no significant difference was observed in adverse events between the 2 groups, and no serious adverse events occurred.

The results on the efficacy of donepezil for post–COVID-19 condition were negative. Fatigue is the most common symptom in post–COVID-19 condition, and in its severe form, it prevents those affected from returning to work.^21^ However, to the best of our knowledge, there are no effective drugs currently available.^12,30^ This is the first trial, to our knowledge, to apply donepezil to the treatment of post–COVID-19 condition. A small RCT that used donepezil to treat memory impairment in patients with COVID-19 was recently reported^31^; however, its effects on post–COVID-19 condition have not yet been examined.

Although a significant effect was not observed in the overall analysis, it may be possible to identify a subgroup with post–COVID-19 condition for whom donepezil is effective. SITH-1, one of the aforementioned HHV-6 component proteins, has been shown to exacerbate psychiatric symptoms by decreasing both the number of acetylcholine-producing cells and the concentration of acetylcholine in the brain,^15^ and HHV-6 reactivation has been detected in some patients with COVID-19.^19^ Blood samples were collected prior to the administration of donepezil in the present study, and a separate biomarker analysis is underway to identify subgroups for whom donepezil is effective.

Donepezil is an ameliorating agent for dementia symptoms, and if it mitigates fatigue and psychiatric symptoms, drug repositioning may be possible. Animal models have shown that donepezil may alleviate psychiatric symptoms, particularly depression, by controlling inflammation through a similar mechanism.^32^ Donepezil has been reported to attenuate depression in some cases of dementia with Lewy bodies.^33^ It has been suggested to act on the sigma-1 receptor in the endoplasmic reticulum and control inflammation in the brain.^34^ If the results of this trial identify subgroups for whom donepezil is effective, we may be able to treat such patients with general psychiatric symptoms.

This trial is novel in that it incorporated a decentralized clinical trial (DCT), taking into account the need for isolation in the acute phase of COVID-19. Our system included e-consent, home visits, and telemedicine, and patients were given the option of having all consultations, apart from the initial one, performed remotely. This system, which decentralizes the clinical trial process, is called a DCT, and is now attracting attention as a form of clinical trial.^35^ The present study was unusual because combined home visits and a DCT have the potential to further facilitate clinical trials for patients requiring acute isolation, such as in this pandemic.

### Limitations

This study has limitations. It was performed as an exploratory phase 2 trial, and it might have led to the study being underpowered to detect significant differences in the outcomes. Thus, a validated phase 3 trial with a larger number of patients is needed, along with valid subgroup identification. In this trial, the intervention was conducted for 3 weeks, and outcomes were assessed 3 and 8 weeks after the initiation of treatment; however, a longer intervention may be necessary. Moreover, the donepezil dose was the same as that used to treat dementia based on the findings of animal studies; however, a higher dose, such as 10 mg, may be effective. Since donepezil was administered to patients without dementia, including young patients, the dose was adapted to that for the treatment of dementia in Japan due to safety considerations. Furthermore, inclusion criteria were changed during the study period, which would have introduced some bias. Fatigue in post–COVID-19 condition is sometimes complicated by general chronic fatigue syndrome or psychiatric disorders, which are difficult to differentiate. In the present study, the attending physician made every effort to exclude these patients by a careful examination; however, some of these patients may still have been included. Furthermore, symptoms should be prolonged at least 1 or 3 months after COVID-19 onset in the definition of post–COVID-19 condition, and we could not identify whether the participants truly had post–COVID-19 condition or not with the inclusion criteria for this study. In addition, although validated assessment tools were used to evaluate these symptoms, they are subjective and may have been affected by the patient’s mental state and other conditions. We used the CFS as the primary outcome measure; however, it might be subjective and might have had an issue of sensitivity and specificity for the symptoms of post–COVID-19 condition. We could not introduce exercise tests because it was necessary to avoid face-to-face evaluations with patients after COVID-19 diagnosis as much as possible due to the pandemic. Future trials should use more objective measures and refine regimens better tailored to the patient population.

## Conclusions

The efficacy of donepezil for post–COVID-19 fatigue and psychological symptoms was not confirmed in a general population in this RCT. In future studies, a biomarker analysis will be conducted to identify subgroups for whom donepezil may be effective.

## Notes

Supplement 1.
Trial Protocol

Supplement 2. **eTable 1.** Medical History and Comorbidities of Participants **eTable 2.** Number of Missing Data in the Full Analysis Set **eTable 3.** Characteristics of the Patients Lost to Follow-Up **eTable 4.** An Absolute Value of Each Outcome in the Full Analysis Set **eTable 5.** An Absolute Value of Each Outcome in the Per-Protocol Set **eTable 6.** Subgroup Analyses Based on Age, Sex, Medical History, and Disease Severity

Supplement 3.
Data Sharing Statement
