# Supplementary material for: Donepezil for Fatigue and Psychological Symptoms in Post–COVID-19 Condition: A Randomized Clinical Trial
Source: JAMA Netw Open. 2025 Mar 17;8(3):e250728. doi: 10.1001/jamanetworkopen.2025.0728 (PMC11915061; doi:10.1001/jamanetworkopen.2025.0728)
Supplement: Supplement 1. — Trial Protocol [file jamanetwopen-e250728-s001.pdf]

trial implementation protocol

Study protocol number: DNPAC-0001

trial subject

Donepezil Drug Repositioning for the Treatment of COVID-19 Sequelae

-Repositioning to Psychiatric Symptom Therapeutics

Kensuke Nakamura, Clinical Trial Coordinator

Associate Professor, Department of Emergency Medicine, Teikyo University Hospital, Teikyo University School of Medicine, Department of Emergency Medicine, Center for Advanced Emergency Medical Care

2-11-1 Kaga, Itabashi-ku, Tokyo 173-8606, Japan

TEL:03-3964-1211 FAX:03-3964-9849

E-mail:k knakamura-tky@umin.ac.jp

Edition: Version 3.0

Creation Date: Feb. 2023

Confidentiality Statement

This material contains information that is provided only to those directly involved in the clinical trial and others. Transfer, reproduction, publication, or disclosure to third parties of this material without prior consent of the coordinating investigator (Clinical Trial Coordinating Secretariat) is prohibited.

Revision History

Version 1.0	First edition established
Version 1.1	See the change contrast table (Version 1.0 -> Version 1.1).
Version 2.0	See Change Contrast Table (Version 1.1 -> Version 2.0).
Version 2.1	See Change Contrast Table (Version 2.0 -> Version 2.1).
Version 3.0	See Change Contrast Table (Version 2.1 -> Version 3.0).

4 Summary of Clinical Trial Protocol

Clinical Trial Coordinating Physician	Kensuke Nakamura
investigational new drug provider	na
trial subject	Donepezil Drug Repositioning for the Treatment of COVID-19 Sequelae - Repositioning for the Treatment of Psychiatric Symptoms
investigational new medical institution	(see Appendix)
phase	Phase 2
Clinical Trial Period	October 2022 - June 2023
Objective.	To test the efficacy of reducing fatigue and depression after COVID-19 infection in COVID-19 patients with fatigue symptoms
Primary Endpoint	Change in Chalder Fatigue-11 score from the previous observation period to 3 weeks after administration of the study drug and absolute values
secondary endpoint	Change in Chalder Fatigue-11 score from the pre-trial observation period to 8 weeks after administration of the study drug and absolute values<br>Change in HADS-A,D scores and absolute values from the pre-observation period to 3 and 8 weeks after administration of the study drug<br>Change in IES-R scores and absolute values from the pre-observation period to 3 and 8 weeks after administration of the study drug<br>Change in EQ5D scores and absolute values from the pre-observation period to 3 and 8 weeks after administration of the study drug<br>Change in PHQ-9 scores and absolute values from the pre-observation period to 3 and 8 weeks after administration of the study drug<br>Symptomatic symptoms including fatigue at 3 weeks and 8 weeks after administration of investigational drug<br>Adverse Events<br>Medication compliance rate
Other evaluation items	Biomarker Evaluation in Blood Testing
Clinical Trial Design	A Multicenter Double-Blind Randomized Controlled Trial
Target number of subjects	120 cases (donepezil group: 60 cases, placebo group: 60 cases)
target disease	Patients with mild to moderate COVID-19
Selection and Exclusion Criteria	**Selection Criteria**<br>1) Patients who are at least 20 years old and less than 75 years old at the time of obtaining consent.

	2) Patients with COVID-19 infection who had upper respiratory tract symptoms or fever or cough in the acute phase.<br>3) Patients with a dichotomous score ≥4 in the Chalder Fatigue Score-11 assessment in the preobservation period.<br>(4) Patients with confirmed positive COVID-19 infection by antigen or PCR test and within 52 weeks from the onset of COVID-19 to randomization.<br>(5) Patients whose consent has been obtained from the patient.<br>[Exclusion Criteria<br>(1) Patients who have been previously diagnosed or suspected to have a psychiatric disorder that falls under F0 to F3 of ICD-10, chronic fatigue syndrome, or other chronic illness associated with fatigue, and patients whose new-onset depressive symptoms are not caused by COVID-19 but by the onset of a psychiatric disorder that falls under F0 to F3 of ICD-10, and who have been diagnosed with a psychiatric disorder that falls under F0 to F3 of ICD-10 The investigator believes that the new onset of depressive symptoms is not due to COVID-19 but is due to the onset of a psychiatric disorder that falls under ICD-10 F0-F3. (See Appendix 1 for ICD-10 classification.)<br>(2) Patients who are likely to start maintenance dialysis or dialysis during the study period.<br>3) Patients with Child-Pugh Classification C cirrhosis.<br>4) Patients with COPD, interstitial pneumonia, or other respiratory diseases (Hugh-Jones classification ≥ II).<br>5) Patients with heart failure (NYHA≥2).<br>6) Patients who are unable to answer the questionnaire in person (including those with cognitive decline).<br>7) Patients with influenza infection.<br>8) Patients already taking donepezil or anticholinesterase inhibitors.<br>9) Patients who are allergic to any component of the investigational drug.<br>10) Patients with a history of hypersensitivity to piperidine derivatives.<br>11) Patients who have participated in the same clinical trial in the past.<br>(12) Patients who do not agree to use contraception by themselves or their partners during the study period. In addition, patients who are pregnant, suspected to be pregnant, or breast-feeding.<br>(13) Other patients who are judged by the investigator (subinvestigator) to be inappropriate as subjects.
investigational new drug	Donepezil and placebo
Dosage and Administration	Donepezil group

	Donepezil 3 mg once daily for 1 week, followed by donepezil 5 mg once daily for 2 weeks.<br>Placebo group<br>Lactose 0.6 g once daily for 1 week, then lactose 1.0 g once daily for 2 weeks.
duration of a drug administration	3 weeks

flowchart

Figure 1 shows the workflow of this clinical trial from obtaining consent to the end of the trial.

Figure 1.

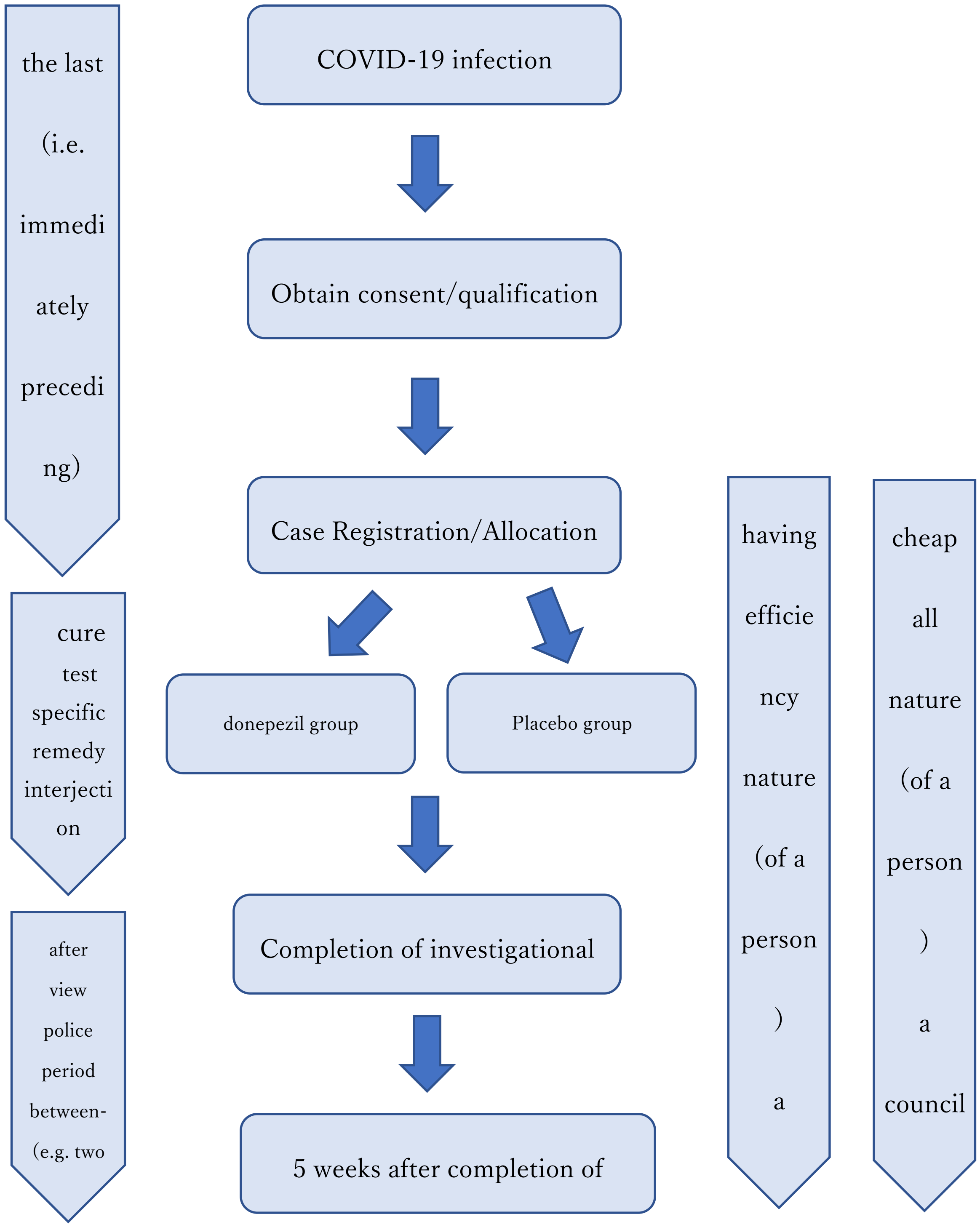

Table of Contents

Revision History ... 1
Summary of Clinical Trial Protocol ... 2
Flowchart ... 5
List of Abbreviations and Terms ... 10
1 foreword ... 11
1.1 Background and Development ... 11
1.2 Profile of test drug ... 11
1.3 Rationale for Conducting Clinical Trials ... 11
1.4 Risk-Benefit Assessment ... 12
1.4.1 RISK ... 12
1.4.2 BENEFITS ... 12
1.4.3 Considerations ... 12
2 Purpose of the Clinical Trial and Endpoints of the Study ... 13
2.1 Primary objective, primary endpoints and secondary endpoints ... 13
2.1.1 Main Objectives ... 13
2.1.2 Primary endpoint ... 13
2.1.3 Secondary endpoints ... 13
2.2 Other objectives and search items ... 13
2.2.1 Other Objectives ... 13
2.2.2 Search Items ... 14
3 Clinical Trial Design ... 14
3.1 Clinical Trial Design ... 14
3.2 Discussion of Clinical Trial Design ... 14
4 Study Population and Discontinuation Criteria ... 14
4.1 Target Diseases ... 14
4.2 Selection Criteria ... 14
4.3 Exclusion Criteria ... 15
4.4 Discontinuation criteria ... 16
5 Clinical Trial Treatments ... 16
5.1 Test drug and placebo ... 16
5.1.1 Increase in dosage of investigational drug ... 16
5.1.2 Method of allocation of patients to treatment groups ... 16
5.1.3 Blinding and Unblinding Procedures ... 17
5.1.4 Packaging and labeling of investigational drugs ... 17

5.1.5 Storage Conditions for Investigational Drugs 17
5.1.6 Clinical Trial Drug Management 17
5.2 Concomitant medications/adjunctive therapy 18
5.2.1 Concomitant medications/adjunctive therapies 18
5.2.2 Prohibited concomitant medications and prohibited concomitant therapies 18
5.3 Concomitant medications to be taken with caution 18
5.4 Confirmation of medication status 18
6 Rating 19
6.1 Efficacy Rating 19
6.1.1 Chalder Fatigue Scale 19
6.1.2 Hospital Anxiety and Depression Scale (HADS) 19
6.1.3 Impact of Event Scale-Revised (IES-R) 19
6.1.4 European Quality of Life Health Status Questionnarire-5 dimensions 5-level (EQ-5D-5L) 19
6.1.5 Patient Health Questionnaire-9 (PHQ-9) 20
6.1.6 Amount of activity, duration of activity 20
6.1.7 Biomarker Evaluation in Blood Testing 20
6.2 Safety Assessment 20
6.2.1 Subject Background 20
6.2.2 Symptoms associated with COVID-19 infection 20
6.2.3 COVID-19 vaccination history 21
6.2.4 Height/Weight 21
6.2.5 Vital signs 21
6.2.6 Oxygen saturation 21
6.2.7 Clinical examination 21
6.2.8 Columbia Suicide Severity Rating Scale (C-SSRS) (see Appendices 2 and 3) 22
6.3 Assessment of adverse events 22
6.3.1 Definition of Adverse Events 22
6.3.2 Collection of adverse events 24
6.3.3 Adverse Event Reporting and Procedures 24
6.3.4 Overdose 25
6.3.5 Pregnancy 26
6.4 Other Ratings 26
6.5 Adequacy of Measurements 26
7 Clinical Trial Plan 26
7.1 Visit Schedule 26
7.2 Details of the test procedure for each Visit 28

7.2.1 Telemedicine 28
7.2.2 Visits 28
7.2.3 Previous observation period 28
7.2.4 Duration of investigational drug administration 28
7.2.5 At discontinuance 29
7.2.6 Duration of Follow-up and Termination of Clinical Trial 29
8 Statistical Analysis and Sample Size 29
8.1 null hypothesis and alternative hypothesis 29
8.2 Analysis Plan 29
8.2.1 Treatment of Interim Events 29
8.2.2 Analysis of primary objective 29
8.2.3 Analysis of secondary objectives 30
8.2.4 Analysis for other purposes 30
8.2.5 Interim Analysis 30
8.3 Handling of missing data 30
8.4 Randomized 30
8.5 Sample size setting 30
9 Policies on obtaining consent, clinical trial records, data protection, and publication; clinical trial management organization 31
9.1 Approval of Clinical Trials, Information to Patients and Obtaining Consent 31
9.2 Data Quality Assurance 31
9.3 Record 32
9.3.1 Original document 32
9.3.2 Direct access to original documents 32
9.3.3 Retention period for records 33
9.4 Emergency reporting of adverse events 33
9.5 Confidentiality and patient privacy 34
9.6 Clinical Trial Management Organization 34
9.6.1 Clinical Trial Coordinators 34
9.6.2 Medical experts 34
9.7.3 Development of therapeutic biomarkers 34
9.7.4 Clinical trial sites 34
9.7.5 Drug donors for clinical trials 35
9.7.6 Monitoring 35
9.7.7 Audit 35
9.7.8 Registration Office 35

9.7.9 Statistical analysis 35
10 References 35
10.1 Publicly disclosed data 35
10.2 Non-public data 35
11 Attachment .... **Error! Bookmark not defined.**
11.1 Appendix 1 ICD-10 (International Classification of Diseases) .... **Error! Bookmark not defined.**
11.2 Appendix 2 C-SSRS (at initial assessment) .... **Error! Bookmark not defined.**
11.3 Appendix 3 C-SSRS (at 2nd and subsequent evaluations) .... **Error! Bookmark not defined.**

List of abbreviations and terms

assistant language teacher (technical term used in Japan)	Alanine Aminotransferase	alanine aminotransferase
AST	Aspartate Aminotransferase	aspartate aminotransferase
BUN	Blood Urea Nitrogen	urea nitrogen
CRP	C-Reactive Protein	C-reactive protein
HbA1c	Glycated Haemoglobin	glycated hemoglobin
LDH	Lactate Dehydrogenase	lactate dehydrogenase

1 foreword

1.1 Background and Development

COVID-19 sequelae occur in about 30% of COVID-19 patients, with the most frequent symptoms being malaise and depression; sequelae appear even when the acute phase of COVID-19 symptoms are mild and occur at high rates even in well vaccinated individuals. The number of patients with sequelae has been increasing along with the increase in the number of patients due to the outbreak of the Omicron strain. The pathogenesis of COVID-19 is unknown, and the definition and classification of the disease have not yet been established. It is known that conventional antidepressants and anti-inflammatory drugs have little therapeutic effect on fatigue and depressive symptoms, and the development of effective treatments is still awaited.

1.2 Test drug profile

The drug to be developed is an acetylcholinesterase inhibitor donepezil hydrochloride (Aricept® Eisai), which is currently widely used as a treatment for dementia, and is intended to be converted into a treatment for fatigue and depression that are sequelae of COVID-19.

In addition to COVID-19 sequelae, donepezil, the drug under development, has the potential to treat malaise and depression caused by viruses that damage the olfactory bulb, and may have applications in malaise, depression, and neuropathy caused by human herpesvirus 6 and influenza viruses.

As for competing drugs with the same mechanism of action, the relationship between COVID-19 sequelae and decreased acetylcholine production in the brain is itself a new discovery, so drug development from the perspective of this research plan has not been published.

1.3 Rationale for Conducting Clinical Trials

Recently, SITH-1 protein of human herpesvirus 6 (HHV-6), which latently infects the olfactory bulb, was found to cause depression by inducing olfactory bulb damage; when SARS-CoV-2 protein, the causative virus of COVID-19, was expressed in the nasal cavity, the olfactory bulb was damaged, The animals exhibited malaise and depressive symptoms, and the researchers succeeded in creating an animal model of COVID-19 sequelae characterized by malaise and depressive symptoms, and found that the SARS-CoV-2 protein was the causative protein of COVID-19 sequelae.

In this animal model, a decrease in acetylcholine-producing cells in the medial septal field and diagonal zone occurred, indicating the possibility of treatment with donepezil hydrochloride, which acts to increase acetylcholine.

Administration of donepezil to this model mouse improved the time of the weighted forced swimming test (an index of malaise) and the immobility time in the tail-suspension test (an index of depression). Spontaneous locomotor activity, a marker of fatigue and depression, also improved.

It was also confirmed that oral administration of donepezil at the usual daily dosage (5 mg or 10 mg) used for dementia patients in human equivalent is sufficient to treat inflammation in the brain, which is thought to be the cause of fatigue and depression. Furthermore, the molecular mechanism of this inflammation in the brain and its improvement by donepezil was found to be the decreased expression of ZFP36, a known mRNA degrading factor of inflammatory cytokines, and its

restoration by donepezil. This finding may be important for elucidating the pathogenesis of COVID-19 encephalopathy.

The development of a treatment for COVID-19 sequelae is urgently needed because of the huge number of patients in addition to the lack of established treatment methods. This R&D project will divert donepezil, which has been confirmed to be safe, at the default dosage, and should address the urgency expected of a therapeutic agent.

1.4 risk-benefit assessment

At this time, there is no established treatment for COVID-19 sequelae, and it is known that usual antidepressants and anti-inflammatory drugs have little therapeutic effect on fatigue and depression.

1.4.1 risk

Donepezil is widely used as a treatment for dementia and has an established safety profile. However, this study is being conducted on a patient population different from that for which the drug is approved, and risks will be discussed based on the results of this study. Therefore, the safety of the subjects will be ensured through adequate monitoring of the subjects throughout the study period.

The fact that patients in this study are mild to moderately ill with COVID-19 infection and that inclusion is after antipyretic therapy will not affect treatment in the acute phase of COVID-19 care. In addition, the use of antipyretics outside of the preanalytic evaluation and other treatment restrictions related to COVID-19 infection will not be implemented after inclusion, thus minimizing the risk.

1.4.2 benefit

The benefits obtained from the administration of donepezil to patients with COVID-19 sequelae are based on predictions from nonclinical results in animal models of COVID-19 sequelae. Donepezil administration may improve inflammation in the brain, which is thought to be the cause of malaise (fatigue) and depression.

1.4.3 consideration

Based on the results of nonclinical studies, donepezil may improve fatigue and depression in patients with COVID-19 sequelae. A placebo subject group is needed to achieve the primary objective of this study. Current knowledge indicates that usual antidepressants and anti-inflammatory drugs have little therapeutic effect, and the possibility of concomitant use with drugs other than donepezil makes placebo administration ethically justifiable.

Although there is a possibility of unknown adverse events in this study, the risks to patients participating in this study are minimal because the mechanism of action of donepezil is well understood and patients will be carefully monitored throughout the study period. We believe that the risks to patients participating in this trial are minimal and that the potential benefits of demonstrating efficacy in the treatment of COVID-19 sequelae are reasonable.

2 Clinical Trial Objectives and Endpoints

2.1 Primary objective, primary endpoints and secondary endpoints

2.1.1 Main Objective

The goal is to conduct a Phase II investigator-initiated clinical trial using donepezil, whose safety has already been confirmed, administered in the usual way, to verify its efficacy in reducing fatigue and depression after COVID-19 infection in COVID-19 patients with fatigue symptoms, to obtain POC, to out-license to a company, and to proceed to Phase III clinical trials. The goal is to obtain POC and to proceed to the out-licensing to companies and Phase III clinical trials.

2.1.2 Primary Endpoint

Change in Chalder Fatigue-11 score from the previous observation period to 3 weeks after administration of the study drug and absolute values

2.1.3 secondary endpoint

The following items shall be set as secondary evaluation items

Change in Chalder Fatigue-11 score from the pre-trial observation period to 8 weeks after administration of the study drug and absolute values

Change in HADS-A,D scores and absolute values from the pre-observation period to 3 and 8 weeks after administration of the study drug

Change in IES-R scores and absolute values from the pre-observation period to 3 and 8 weeks after administration of the study drug

Changes and absolute values of EQ5D score from the pre-observation period to 3 and 8 weeks after administration of the study drug

Change in PHQ-9 scores and absolute values from the pre-observation period to 3 and 8 weeks after administration of the study drug

Symptomatic symptoms including fatigue at 3 weeks and 8 weeks after administration of investigational drug

Adverse Events

Medication compliance rate

2.2 Other objectives and search items

2.2.1 Other Objectives

A biomarker search will be conducted to determine which patients will benefit from donepezil and to create a basis for the development of companion diagnostics for the selection of therapies that will be useful in actual treatment. Biomarkers may be used for research other than biomarker development for this clinical trial. Consent for use outside of this clinical trial will be obtained from the subject in advance. In addition, we will measure the number of steps taken using Yamasa's pedometer for exploratory purposes.

2.2.2 search item

Activity volume and duration at 1, 2, and 3 weeks after administration of the investigational drug

Biomarker Evaluation in Blood Testing

3 Clinical Trial Design

3.1 Clinical Trial Design

This is a phase 2, randomized, double-blind, investigator-initiated clinical trial (investigator-initiated clinical trial) of donepezil in patients with mild to moderate COVID-19 with fatigue symptoms, ages 20 to <75 years, with symptoms of COVID-19, and within 52 weeks of onset of symptoms. patients aged 20 to less than 75 years with COVID-19 symptoms and a Chalder Fatigue Score-11 dichotomous score of ≥4 within 52 weeks of onset of symptoms. Participation in the study will last for 8 weeks, and subjects who consent and are eligible will receive standard treatment for COVID-19 (or none if no treatment is needed) and will be randomized in a 1:1 ratio to either the donepezil or placebo group. The subjects will then receive either donepezil or placebo for 3 weeks in a double-blind fashion, with follow-up assessments at 3 weeks and 8 weeks after the start of the study drug. The number of subjects will be 60 in each group, for a total of 120 subjects.

The test drug will be donepezil (Aricept) powder purchased from the market under the technical guidance of Eisai Inc. and the control drug will be lactose powder as placebo.

3.2 Considerations for Clinical Trial Design

The purpose of this trial is to evaluate the efficacy of donepezil in patients with COVID-19 sequelae.

After COVID-19 positivity is confirmed by antigen or PCR and eligibility is confirmed with standard therapy, patients will be started on donepezil or placebo. Patients will be started on a low dose (3 mg donepezil) for the first week of treatment to determine if any gastrointestinal adverse events occur. If no adverse events attributable to donepezil are observed, the dose is increased (5 mg donepezil) for 2 weeks. The design of this study is in accordance with the donepezil package insert, and we believe that there are no safety issues for patients. Based on the results of basic research, it is expected that sufficient efficacy can be determined during the post-dose escalation period. Outcome symptom evaluation will also be conducted after 8 weeks of investigational use for long-term confirmation as a post-dose evaluation.

Based on the above, we believe that this is an appropriate design for this clinical trial.

4 Study Population and Discontinuation Criteria

4.1 target disease

Patients with mild to moderate COVID-19 with malaise symptoms.

4.2 criterion (criteria) for selection

1) Patients between 20 and 75 years of age at the time consent is obtained.
2) Patients with COVID-19 infection who had upper respiratory tract symptoms or symptoms of fever or cough in the acute phase.

3) Patients with a bivalent score ≥ 4 on the Chalder Fatigue Score-11 assessment in the previous observation period.

4) Patients with confirmed positive COVID-19 infection by antigen or PCR test and within 52 weeks from COVID-19 onset to randomization.

5) Patients whose consent has been obtained from the patient.

<Rationale for setting

(1) The study was conducted in adults, and criteria were established to exclude patients in the later stages of life.

(2) to (4) Criteria were established for inclusion of subjects who were eligible for this study.

(5) Criteria were established for inclusion of patients who gave consent.

4.3 exclusion criteria

(1) Patients who have been previously diagnosed or suspected to have a psychiatric disorder that falls under F0 to F3 of ICD-10, chronic fatigue syndrome, or other chronic illness associated with fatigue, and patients whose new-onset depressive symptoms are not caused by COVID-19 but by the onset of a psychiatric disorder that falls under F0 to F3 of ICD-10, and who have been diagnosed with a psychiatric disorder that falls under F0 to F3 of ICD-10 The investigator believes that the new onset of depressive symptoms is not due to COVID-19 but is due to the onset of a psychiatric disorder that falls under ICD-10 F0-F3.(See Appendix 1 for ICD-10 classification.)

2) Patients who are likely to start maintenance dialysis or dialysis during the study period.

3) Patients with Child-Pugh Classification C cirrhosis.

4) Patients with COPD, interstitial pneumonia, or other respiratory diseases (Hugh-Jones classification $\geq$ II).

5) Patients with heart failure (NYHA$\geq$2).

6) Patients who are unable to answer the questionnaire in person (including those with cognitive decline).

7) Patients with influenza infection.

8) Patients already using donepezil or anticholinesterase inhibitors.

9) Patients who are allergic to any component of the investigational drug.

10) Patients with a history of hypersensitivity to piperidine derivatives.

11) Patients who have participated in the same clinical trial in the past.

(12) Patients who do not agree to use contraception by themselves or their partners during the study period. In addition, patients who are pregnant, suspected to be pregnant, or breast-feeding.

(13) Other patients who are judged by the investigator (subinvestigator) to be inappropriate as subjects.

<Rationale for setting

1), 11) Criteria were established to properly evaluate this clinical trial.

(2)-7) Criteria were established to exclude subjects with serious complications.

8) Criteria were established to exclude infections similar to COVID-19.

9) – 10), 12), 13) Criteria were set to ensure the safety of the subjects.

4.4 Criteria for discontinuance

(1) When a subject declines to participate in a clinical trial or withdraws his/her consent.

(2) If an event occurs that makes it difficult to continue the clinical trial.

(3) If the subject is found to be outside the scope of the clinical trial after enrollment.

(4) If the entire study is terminated.

(5) When the investigator(s) deems it appropriate to discontinue the study for other reasons.

5 Clinical Trial Treatments

5.1 Test drug and placebo

The only investigational drugs used in this study are donepezil (test drug) and placebo (control drug).

After COVID-19 diagnosis, both groups will receive standard treatment (or none if no treatment is needed), and then either donepezil or placebo for 3 weeks in a double-blind fashion according to allocation. If a patient misses a dose, the dose will be taken the following day. If the patient visits the clinic or telemedicine point 3 weeks after the start of treatment and before the end date of medication, the patient will be instructed to finish 5 mg of donepezil or placebo.

Donepezil group

Donepezil 3 mg once daily for 1 week, followed by donepezil 5 mg once daily for 2 weeks.

Placebo group

Lactose 0.6 g once daily for 1 week, then lactose 1.0 g once daily for 2 weeks.

5.1.1 Increase in dose of investigational drug

The investigator will confirm the presence or absence of adverse events by visiting the subject one week after administration (or by online examination if the subject cannot visit the hospital) according to "7.1Visit Schedule". If no adverse events are observed or if the adverse events are tolerable, the investigator will instruct the subject to increase the dose of the study drug and note this in the medical record and source documents. If an unacceptable adverse event is observed, a decision will be made as to whether or not the subject can continue taking the investigational drug. If an unacceptable adverse event is observed in a patient who has undergone online medical care, the patient will be asked to come to the hospital and necessary measures will be taken to determine whether or not the subject can continue taking the medication. If it is determined that the medication cannot be continued, the study will be discontinued and the discontinuation will be noted in the source documents.

5.1.2 Method of allocation of patients to treatment groups

After COVID-19 diagnosis, both groups will receive standard treatment (or none if no treatment is needed) and eligible subjects will be assigned to donepezil or placebo in a 1:1 ratio.

Subjects will be prescribed the investigational drug after the investigator (subinvestigator) determines that they are eligible.

The investigator or collaborator registers eligible patients in Bellsystem24's Web Registration System for Limited Function (hereinafter referred to as "Web Registration System"), and the investigator or collaborator prescribes the numbered investigational drug for use to the subject.

5.1.3 Blinding and unlocking procedures

5.1.3.1 blinding

The donepezil and placebo groups will be double-blind and will remain blinded until the completion of the study for all subjects. The investigational drug will be packaged in aluminum and the appearance of the study drug and placebo will be indistinguishable.

5.1.3.2 Unblinding and unlocking procedure

The investigator may unblind the subject through the web-based registration system in an emergency. This must only be used in emergency situations where the investigator must identify the investigational drug in order to provide appropriate medical treatment to the subject or to ensure the safety of the subject. The reason for unblinding shall be documented in the source documents and the case report form.

The investigator will inform the coordinating investigator and the coordinating office about the unblinded subjects.

5.1.4 Packaging and labeling of investigational drugs

The investigational drug will be delivered to each medical institution from SD Logistics Co. Packaging and labeling of the study drug and placebo will be conducted in accordance with the principles of Good Manufacturing Practice (GMP) for investigational new drugs.

For details on packaging and labeling, refer to the "Procedures for the Management of Investigational Drugs Used in Clinical Trials.

5.1.5 Storage conditions for investigational drugs

Investigational drugs should be stored at room temperature in a secure and controlled environment. Access to the investigational products is limited to the investigator and authorized site personnel.

Any deviation from the temperature range of the investigational drug should be reported promptly in accordance with the procedures for the management of investigational drug use.

5.1.6 Clinical Trial Drug Management

Information regarding the prescription and return of investigational drugs for each subject in the study shall be recorded on the Investigational Drugs Control Chart, which shall be used as the source document throughout the study period. Information on loss, damage (breakage, loss), non-use, and disposal by the subject of the investigational drug should also be recorded in the chart. All records related to the transfer of investigational new drugs should be available for review at any time throughout the clinical trial period.

5.2 Concomitant Drugs and Concomitant Therapy

Information on concomitant medications and concomitant therapies will be collected from the time of obtaining consent to the end of the clinical trial.

5.2.1 Concomitant medications/adjunctive therapy

Drugs other than concomitant use prohibited drugs, drugs other than concomitant use cautioned drugs, and treatments other than concomitant use prohibited therapies may be taken or administered at the discretion of the investigator (subinvestigator). However, only ultra-short-acting sleeping pills (zolpidem, zopiclone, eszopiclone) may be used.

5.2.2 Prohibited drugs and prohibited therapies

<Combination Prohibited Drugs

Antiepileptic, antipsychotic, antidepressant, anxiolytic, central stimulant, and antiparkinsonian drugs,

Use of antipyretics within 24 hours prior to conducting the preobservation period outcome symptom survey

<Combination Prohibited Therapy

Repetitive transcranial magnetic stimulation (rTMS) therapy, nasopharyngeal abrasion therapy

Acupuncture and moxibustion do not fall under the category of concomitant prohibited therapy, but if they are practiced, they should be noted in the case report.

5.3 drug to be used in combination with

The following drugs should be used with caution, as they have been reported to increase or decrease the effect of donepezil due to drug interactions. The investigator should decide whether or not to use these drugs.

Suisamethonium chloride hydrate

Choline activator

Cholinesterase inhibitor

Drugs that inhibit or induce CYP3A

Central anticholinergic agent

Atropine-type anticholinergic agent

Non-steroidal anti-inflammatory analgesics

5.4 Confirmation of medication status

Unused investigational drugs, empty boxes, and empty bags will be collected at the 3-week visit after the start of administration to check the medication status. If the patient is unable to visit the clinic, the medication will be collected by mail.

If the medication compliance rate is less than 80%, it should be noted in the source documents as a deviation.

6 evaluation

6.1 Evaluating Efficacy

Chalder Fatigue Scale, Hospital Anxiety and Depression Scale (HADS), Impact of Event Scale-Revised (IES-R), and European Quality of Life Health Status Questionnarire-5 dimensions 5-level (EQ-5D-5L), the assessment items are listed in Appendix 2.

6.1.1 Chalder Fatigue Scale

The Chalder Fatigue Scale is an 11-item rating scale created to measure the severity of fatigue※1 . Each item is rated on a 4-point scale: none (0 points), not much (1 point), more than usual (2 points), and very much (3 points) [only the item on memory is better than usual (0 points), the same as usual (1 point), worse than usual (2 points), and very much worse than usual (3 points)]. Subjects are asked to select the most appropriate response for each of the 11 items.

Since a binary score is employed in this experiment, scores of 0 and 1 are counted as score 0, and scores of 2 and 3 are counted as score 1.

6.1.2 Hospital Anxiety and Depression Scale (HADS)

The HADS is a simple method of assessing mental anxiety and depression and consists of a 14-item questionnaire. Each item consists of alternating anxiety and depression questions, each of which is rated on a 4-point scale. Subjects are asked to select the most appropriate response to each of the 14 items. The investigator assigns a score to anxiety and depression, respectively, according to the HADS score distribution table.

6.1.3 Impact of Event Scale-Revised (IES-R)

The IES-R is a self-administered questionnaire developed by Weiss et al. in the United States as a revised version of the former IES (Horowitz et al, 1979) to measure posttraumatic stress symptoms. The old IES consisted of 15 items (7 intrusion symptoms and 8 avoidance symptoms), but the IES-R adds 6 items for hyperarousal symptoms and further divides the sleep disturbances of the old version into 2 items, difficulty falling asleep and mid-awake, for a total of 22 items.

The IES-R can easily measure PTSD-related symptoms in a wide range of trauma survivors, from disasters to individual victims, and is already widely used in Japan for cross-sectional surveys, symptom follow-up, and screening purposes.

For each item, subjects will be asked to rate on a 5-point scale: not at all, a little, moderately, quite a bit, and very much. Subjects are asked to select the most appropriate response for each of the 22 items.

6.1.4 European Quality of Life Health Status Questionnarire-5 dimensions 5-level (EQ-5D-5L)

The EQ-5D-5L consists of two pages: the descriptive part of the EQ-5D and the EuroQol Visual Assessment Scale (VAS) part. The descriptive part consists of five items: degree of mobility, personal care, usual activities, pain/discomfort, and anxiety/blockiness. Each item is rated on a 5-point scale: no problem, somewhat problematic, standard problematic, fairly problematic, and very problematic. Subjects are asked to indicate their health status by checking the box next to the most appropriate description for each of the five items. The subject records his/her self-assessment of his/her health

status by means of the "best imaginable health status" and "worst imaginable health status. This VAS can be used as a quantitative value of health status reflecting the subject's own judgment.

6.1.5 Patient Health Questionnaire-9 (PHQ-9)

The PHQ-9 is a questionnaire designed to screen for the presence and severity of depression. It consists of nine items on "depressed mood" and "loss of interest" in the last two weeks. Mood" and "Decrease or loss of interest" for the last 2 weeks. Each item is rated on a four-point scale: not at all, a few days, more than half of the time, and almost every day. If even one of the nine items applies to the patient, the effect on work, housework, and interpersonal relationships is rated on a four-point scale: not at all difficult, somewhat difficult, difficult, and extremely difficult.

Instruct the subjects to check the checkboxes for the applicable items.

6.1.6 Amount of activity, duration of activity

A Yamasa pedometer (model number: TM-510) will be used to measure the number of steps taken. The device will be worn daily from the start of treatment until 3 weeks after the start of treatment. The device should be worn daily from the time the patient wakes up to the time the patient goes to bed, if possible.

The next day, the number of steps taken is recorded on the medication logbook for the previous day. Using an electronic medication logbook (Viedoc Me), the subject will take a picture of the step count screen and upload the image to the electronic medication logbook.

Procedures for electronic medication logbooks are specified separately.

6.1.7 Biomarker Evaluation in Blood Testing

Biomarkers will be performed during the preobservation period.

Biomarkers will be collected by SRL Corporation. Procedures for blood collection and pickup will be determined separately.

6.2 Safety Assessment

6.2.1 Subject Background

During the preanalytic period, the following background information will be checked: sex, age, date of birth, date of onset of COVID-19, complications and history, and concomitant medications/adjunctive therapies. However, the date of birth will be entered only in the Web registration system.

6.2.2 Symptoms associated with COVID-19 infection

The presence and severity of the following symptoms associated with COVID-19 infection will be confirmed during the pre-treatment observation period, 3 weeks after the start of treatment, and 8 weeks after the start of treatment. The severity will be evaluated according to "6.3.1.4 Severity of adverse events".

… cough

sputum

Heat generation

Taste disorder

Anorexia

Other

For other items, events that the investigator (sub)investigator determines should be reported should be described in the source documents and the case report form.

6.2.3 COVID-19 vaccination history

Confirm the number of COVID-19 vaccine doses, the date of the last dose, and the type of last vaccine during the previous observation period.

6.2.4 Height and Weight

Measure height and weight during the preobservation period. Record height and weight to one decimal place.

6.2.5 vital signs

The measurements will be performed during the pre-treatment observation period, 3 weeks after the start of treatment, and 8 weeks after the start of treatment. After 3 weeks and 8 weeks of treatment, measurements will be taken only in subjects who come to the hospital. Vital signs include temperature, pulse rate, respiratory rate, systolic and diastolic blood pressure.

6.2.6 oxygen saturation

The measurements will be performed during the pre-treatment observation period, 3 weeks after the start of treatment, and 8 weeks after the start of treatment. After 3 weeks and 8 weeks of treatment, measurements will be taken only in subjects who come to the hospital. Measured with a pulse oximeter.

6.2.7 clinical examination

The following laboratory tests will be performed at the patient's own institution during the pre-treatment observation period, 3 weeks after the start of treatment, and 8 weeks after the start of treatment. Clinical examinations at 3 weeks and 8 weeks after the start of treatment will be performed at the patient's request or at the physician's discretion. Since these tests are performed to ensure subject safety, there is no problem in confirming the test results after randomization.

**(data) item**	Peripheral white blood cell count, neutrophil fraction, lymphocyte fraction, hemoglobin level, platelet count, albumin, AST, ALT, LDH, HbA1c, CRP, BUN, creatinine

For items not listed above, the investigator will perform additional tests as necessary.

In addition, women of childbearing potential should undergo a serum or urine pregnancy test during the preclinical observation period. Patients may be assigned after pregnancy test results are confirmed. Women of childbearing potential are defined as

1) At some point in time, the patient experiences first menstruation.

(2) Has not undergone hysterectomy or bilateral oophorectomy.

(3) Women who are not more than 24 months post spontaneous menopause (i.e., menstruated in the past 24 months).

6.2.8 Columbia Suicide Severity Rating Scale (C-SSRS) (see Appendices 2 and 3)

Donepezil hydrochloride is a drug that acts on the central nervous system. (), it is also essential to confirm the presence or absence of suicidal ideation and suicide attempts.

For this evaluation, the investigator(s) will receive appropriate training prior to conducting the subject evaluation.

If the patient answers "Yes" to either Questionnaire 1 or 2 of the C-SSRS for suicidal ideation during the pre-treatment observation period, the investigator will consult a psychiatrist and allow the patient to participate in this study only if the psychiatrist determines that the patient is not at risk for suicide. If the patient answers "Yes" to either Questionnaire 1 or 2 for suicidal ideation after 3 or 8 weeks of treatment, the investigator will consult a psychiatrist and make a decision to continue or discontinue the study only when the psychiatrist determines that the patient is not at risk for suicide. If the psychiatrist determines that there is a risk of suicide, the study will be terminated. In addition, if necessary, the adverse event should be considered as an adverse event.

6.3 Adverse Event Assessment

6.3.1 Definition of adverse events

An adverse event is any unwanted or unintended illness or symptom (including abnormal laboratory values) that occurs in a subject receiving an investigational drug, regardless of whether or not it is causally related to the investigational drug. The term "adverse event" means any unwanted or unintended illness or symptom (including abnormal laboratory test results) that occurs in a subject who is administered an investigational new drug, regardless of whether or not it is causally related to the investigational new drug. Adverse event" means an adverse event for which there is at least a reasonable possibility of a causal relationship with the investigational drug and a causal relationship cannot be ruled out.

6.3.1.1 adverse event

Adverse events shall be as follows When multiple clinical symptoms or signs (including abnormal laboratory values) occur in association with one adverse event (disease), they should be combined together as a single adverse event in the case report in principle. If the investigator does not draw blood 3 weeks after the start of treatment or 8 weeks after the start of treatment for reasons such as inability to come to the hospital, the investigator will determine the adverse event based on the subject's condition and the presence or absence of symptoms.

Abnormal laboratory values or other safety tests that are considered clinically significant in the medical or scientific judgment of the patient and that have been observed since the time consent was obtained.

Aggravation of an event (symptom) that has already been chronically or intermittently observed.

Signs, symptoms, or sequelae of suspected drug interactions

The investigator determines that the event should be treated as an adverse event.

6.3.1.2 Serious adverse events

Serious adverse events shall be as follows

(1) Death

(2) Obstacles

(3) Cases that may lead to death

(4) Cases that may lead to disability

(5) Cases requiring hospitalization or extended hospitalization at a hospital or clinic for treatment

6) Cases that are as serious as those listed in 1) through 5)

(7) Congenital diseases or anomalies in later generations

The "hospitalization" in 5) does not include hospitalizations (e.g., scheduled surgeries and examinations) for the sole purpose of performing therapies or examinations that were planned prior to participation in the clinical trial during the clinical trial. (However, any new occurrence during such hospitalization shall be treated as an adverse event.)

6.3.1.3 Notable Adverse Events

If any of the following adverse events occur as noteworthy adverse events, the investigator should carefully observe and appropriately treat the patient.

- arrhythmia
- Myocardial infarction, heart failure
- Gastrointestinal ulcer, gastrointestinal bleeding, gastrointestinal perforation
- Hepatitis, jaundice
- malignant syndrome
- dyspnea
- acute pancreatitis
- acute renal failure
- Hypersensitivity (rash, itching)
- Anorexia, nausea, vomiting
- Diarrhea, abdominal pain, constipation

6.3.1.4 Severity of adverse events

The investigator (sub)investigator will classify the severity of each adverse event/serious adverse event reported during the clinical trial into one of the following categories.

Mild: Discomfort is minimal, tolerable, and does not interfere with daily activities.

Moderate: Event with definite discomfort that interferes with daily life.

Severe: An event that interferes with daily life.

6.3.1.5 Causal relationship with investigational drug

The investigator shall evaluate the causal relationship between the investigational drug and each adverse event/serious adverse event. The criteria shall be as follows

judgment	Definition.
No relation	The event is clearly caused by a cause other than the investigational drug (e.g., disease, environment, etc.). Or, there is no reasonable time-related relationship between the investigational drug and the event.
Relevant.	The event is time-related to the onset of the event after administration of the investigational drug, and the event attenuates with time after administration of the investigational drug, but recurs or worsens with subsequent re-administration of the investigational drug.

6.3.1.6 Adverse event outcomes

Adverse event outcomes will be judged on the following five levels

return to origin	evaluation criteria
recovery	Adverse events have resolved or returned to normal.
light	Adverse events have not fully recovered, but have largely disappeared or returned to almost normal.
Recovered but with sequelae	Adverse events have recovered to normal, but sequelae remain.
unrecovered	Adverse events are ongoing.
death	The patient died as a result of an adverse event.
unknown	No information is available and outcome is unknown.

6.3.2 Collection of adverse events

Adverse events will be collected at 1, 3, and 8 weeks after administration of the investigational drug. If an adverse event is observed in a subject during the clinical trial, the investigator (subinvestigator) will immediately take appropriate medical measures to ensure the subject's safety and inform the subject if treatment for the adverse event is necessary.

If an exacerbation of depressive symptoms is observed, a visit to the investigational site or a consultation with a psychiatrist (subinvestigator) should be conducted.

Adverse events shall be followed up until the subject recovers. However, if the outcome is unrecovered or minor at the end of the trial, follow-up is not required only when the event has stabilized and the investigator (subinvestigator) determines that follow-up is not required, with the reason clearly stated in the source documents.

6.3.3 Adverse event reporting and procedures

6.3.3.1 Serious adverse event reporting

If an adverse event occurs in a subject from the start of administration of the investigational drug until 8 weeks after the start of administration, and the investigator determines that the event is serious, the adverse event information will

be handled according to the following procedures.

Regardless of the causal relationship, the investigator shall report the event as soon as possible (within 24 hours) to the head of the site and to the coordinating investigator in accordance with the "Procedures for Handling Safety Information". The starting date of the reporting deadline shall be the date when the investigator becomes aware of the occurrence of a serious adverse event.

(1) Notification by the investigator coordinating the clinical trial to the investigators at each site

The coordinating investigator shall review the contents of the adverse event report obtained from the investigator and notify the relevant adverse event information to the investigators at each of the other sites.

(2) Consultation between the investigator and coordinating investigator

The investigator at each site checks the contents of the report obtained from the coordinating investigator, consults with the coordinating investigator as necessary, and reports his/her opinion as the investigator (including the necessity of reporting to the Minister of Health, Labor and Welfare) to the coordinating investigator.

(3) Report to the Minister of Health, Labor and Welfare

If the investigator determines that a report to the Minister of Health, Labour and Welfare is necessary, the coordinating investigator shall prepare "Forms 7 and 8" and "Organizing Sheet" and report to the Pharmaceuticals and Medical Devices Agency (hereinafter referred to as the "Agency"). The coordinating physician shall report to the Pharmaceuticals and Medical Devices Agency (hereinafter referred to as the "Agency"). In addition, the coordinating physician shall provide information to Eisai Co.

(6) Report to the head of the medical institution

When a report is made to the Minister of Health, Labour and Welfare regarding a serious adverse event that occurred at another medical institution, the investigator shall report the contents of "Forms 7 and 8" obtained from the coordinating investigator to the head of the implementing medical institution as soon as possible.

(7) Actions to be taken when additional information is obtained

The investigator of the site where the adverse event occurred shall make an additional report to the head of the site as soon as possible and to the coordinating investigator when additional information regarding the event is obtained. The same procedure shall be followed for reporting and notification of such additional information.

6.3.3.2 Information provided by the investigator

The investigator who conducts the clinical trial himself/herself shall promptly inform the head of the site when he/she obtains information on all serious and unpredictable adverse effects, etc., and information that may adversely affect the safety of the subject, affect the conduct of the clinical trial, or change the approval of the investigational review committee regarding the continuation of the clinical trial.

6.3.4 drug overdose

In the event of an overdose of an investigational drug, whether intentional or negligent, the coordinating investigator and the Clinical Trial Coordinating Office should be notified immediately. In case of overdose, the investigator will check

the safety of the subject and decide whether the investigational drug can be continued or not. The occurrence of overdosage and the safety of the subject will be described in the source documents. If a serious adverse event occurs due to overdosage, it should be reported as described in 6.3.3.1 Serious adverse events.

6.3.5 pregnancy

Detailed information will be collected on all pregnancies in female subjects and their male partners from the start of the investigational drug until 8 weeks after the start of treatment. If pregnancy is reported, the investigator will report it according to the same procedure as in 6.3.3.1 Report of Serious Adverse Events.

6.4 Other Evaluations

na

6.5 Appropriateness of measurement items

The measurements to be performed in this clinical trial are biomarker measurements. The biomarker measurements will be conducted to search for biomarkers to determine which patients will benefit from donepezil and to create a basis for the development of companion diagnostics for the selection of therapies that will be useful in actual treatment.

7 trial plan

7.1 Visit Schedule

The schedule for this clinical trial is as follows

<Trial Schedule

Item	pre-observation period	Duration of investigational drug administration[a]			post-observational period	halt time
Implementation period (± tolerance)	Previous observation perioda,b	Dose start datea	Start of Dosing One week laterb	Start of Dosing 3 weeks laterb	Start of Dosing After 8 weeksb	
		one day	7 (-3)	21 (-2 to +1)	56 (±7)	
Eligibility Verification	period	period				
Obtaining Consent	symbol used as a placeholder (either because a number of other words could be used in that position or because of					

	censorship)					
Subject Background	period					
Symptoms associated with COVID-19 infection	period			●	●	
COVID-19 vaccination history	symbol used as a placeholder (either because a number of other words could be used in that position or because of censorship)					
Height and Weight	symbol used as a placeholder (either because a number of other words could be used in that position or because of censorship)					
vital signs	period			●c	●c	
oxygen saturation	period			●c	●c	
clinical examination	0e	(0)e		●d	●d	
Biomarker e	period	(Zero)				
Outcome Symptom Survey f	period			●b	●b	
C-SSRS	period			●	●	
Adverse Event Assessment		●	●	●	●	●
Prescription of drugs for clinical trials		0g				
Recall of investigational drugs				●		●h

Items marked with ○ are to be performed before the start of administration of the investigational drug, and items marked with ● are to be performed after the start of administration of the investigational drug.

a: It is acceptable to conduct the preobservation period and the start date of administration on the same day.

b: Pre-observation period, 1 week after the start of treatment, 3 weeks after the start of treatment, and 8 weeks after the start of treatment are performed on an outpatient basis (if necessary, call in advance to confirm conditions for removal of isolation) or via online clinic.

c: Performed only when the subject comes to the hospital

d: Blood tests at 3 weeks after the start of treatment and at 8 weeks after the start of treatment are optional and performed at the discretion of the patient and the investigator (subinvestigator).

e: Biomarker and pre-trial clinical examinations should be performed as an outpatient or during home visits prior to the administration of the study drug if they cannot be performed during the pre-trial period (including cases where the pre-trial period was performed during the online medical examination).

f: Outcome symptom surveys shall be Chalder Fatigue Scale, HADS, IES-R, EQ-5D-5L, and PHQ-9.

G: The investigational drug will be taken for 3 weeks (21 days) regardless of the date of the patient's visit 3 weeks after the start of administration.

h: The investigational drug will be collected only if it is discontinued during the period of administration of the investigational drug.

7.2 Details of the test procedure for each Visit

7.2.1 remote medical examination

If consent is obtained while the subject is under COVID-19 isolation (including cases in which the subject is a concentrated contact), it is possible to obtain consent remotely. Note, however, that if consent is obtained remotely, a visit to the hospital or a clinic visit will be required for biomarker blood collection and clinical examination during the preanalytic period. If the patient is unable to come to the hospital 1 week after the start of treatment, 3 weeks after the start of treatment, or 8 weeks after the start of treatment, or if there are reasons why the patient is unable to come to the hospital, the investigator (or collaborator) will conduct the items specified in "7.1Visit Schedule" by remote consultation using online medical services.

7.2.2 home visitation (esp. of a patient)

If the items specified in "7.1Visit Schedule" are feasible at the discretion of the investigator (responsible party), the home visit is permitted. However, when conducting home visits, procedures for home visits must be established at each site and the procedures must be followed.

7.2.3 pre-observation period

The investigator obtains consent from the subject. Consent will be obtained in paper form or by eConsent at the discretion of the investigator, depending on the condition of the subject. The investigator will then confirm whether the subject is eligible for the study and include the subject in the clinical trial. The items to be conducted during the pre-observation period will follow "7.1Visit Schedule".

7.2.4 Duration of investigational drug administration

The duration of study drug administration is 3 weeks. During the study drug administration period, subjects will receiveeither donepezil or placebo. The protocol for the dosing period will follow "7.1Visit Schedule".

7.2.5 time of discontinuance

Patients who discontinue during the study period will be followed up either in clinic or via online clinic to perform a Visit at Discontinuation. For patients who discontinue the study drug during the study period and are unable to come to the hospital, the investigational drug remaining at home will be returned to the medical institution under the direction of the investigator (subinvestigator). In addition, when follow-up is conducted by telephone, the investigator will instruct the subject to come to the hospital if necessary if an adverse event is observed in the subject.

7.2.6 Duration of follow-up and completion of the clinical trial

Subjects who have completed the study drug administration period will be followed up at 3 weeks and 8 weeks after the start of the study drug administration in principle in the clinic. If it is difficult to visit the clinic, online follow-up is acceptable. The study will be terminated at the 8-week follow-up.

8 Statistical Analysis and Sample Size

8.1 null hypothesis and alternative hypothesis

The null hypothesis: there is no difference in a priori primary outcome between donepezil and placebo groups for patients with mild to moderate COVID-19 disease.

8.2 analysis plan

The primary endpoints are compared using t-tests and regression models, while other outcomes and observables are compared using t-tests, Wilcoxon tests, $\chi 2$ tests, and regression models.

8.2.1 Treatment of Interim Events

In this study, the use of concomitant medications prohibited by the study protocol, treatment discontinuation, pregnancy, and death will be treated as intermediary events and considered in the analysis.

8.2.2 Primary Objective Analysis

8.2.2.1 Sensitivity Analysis

Both intention-to-treat and per-protocol analyses will be performed.

8.2.2.2 subgroup analysis

Subgroup analysis by age, gender, history, and severity of disease.

8.2.2.3 Additional Analysis

Not planned at this time.

8.2.3 Analysis of secondary objectives

Secondary endpoints are compared using t-tests and regression models. Other outcomes and observations are compared using t-tests, Wilcoxon tests, $\chi 2$ tests, and regression models. Mixed effects models and generalized estimating equations will be used for time series data.

8.2.4 Other Purpose Analysis

The outcome is defined as donepezil response, and multivariate regression models and Lasso regression are used to search for characteristic biomarkers.

8.2.5 Interim Analysis

No interim analysis will be performed in this trial.

8.3 Handling of Missing Data

Identify intermediate events or missing measurements, and for those that can be followed up, address the missing measurements by referring to the hospital chart or by asking the patient. If missing data cannot be filled in, supplement as necessary.

8.4 randomization

Block randomization will be employed in this trial.

8.5 Sample size setting

In a double-blind randomized controlled trial (n=200) with CFQ-11 as the outcome of COVID-19 by Rathi et al. the mean CFQ-11 of patients in the placebo group on days 8, 11 and 14 of COVID-19 onset is 21.75, 20.55 and 19.91, respectively, and therefore the patients participate CFQ-11 at timing, i.e., the baseline CFQ-11 value was set at 20 (Rathi A et al. Medicines (Basel) 2021;8(9)47). In this study, CFQ-11 values decreased from baseline to day and time in both groups from day 0. The mean CFQ-11 value of 128 COVID-19 patients months after onset was 15.8±5.9 (Townsend L et al. PLOS ONE 2020;15(11):e0240784), and this figure was similar in another outpatient study of 458 patients (Stavem K et al. Int J Environ Res Public Health. 2021;18(4):2030). Based on these studies, the mean CFQ-11 score after one month in the non-treated group was set at 15 ± 5 and the amount of decline at 5, and the expected treatment effect was set at a 3-point reduction in minimal important difference from previous studies (Nordin Asa et al. BMC Med Res Methodol. 2016;16:62). Assuming a dropout rate of about 25%, we set α error = 0.5 and β error = 0.20. Based on these values, we set the number of patients to 120, 60 in each group.

[Sample size design (modified: February 12, 2023)

At the February 9, 2023 study group meeting, it was determined that the 8th group of COVID-19 was winding down, and as a result, it would be difficult to collect the above sample size by the scheduled time. As a result, it was determined that

it would be difficult to collect the above sample size by the scheduled date. Therefore, the sample size calculation was performed again due to the revised patient inclusion criteria. The revised inclusion criteria were changed from "within 21 days of disease onset" to "within 52 weeks of COVID-19 onset to randomization," so CFQ-11 scores obtained from previous publications at approximately one year after COVID-19 onset were used. A study in Ireland that followed patients hospitalized with COVID-19 for approximately one year showed a CFQ of 16.7 ± 5.6 after a mean follow-up of 430 days (O'Brien K, et al. Respir Res. 2022 23(1):115), a Spanish population-based stdy study found a CFQ-11 of 8±3 after a mean of 36 weeks (Jimeno-Almazan A et al. Intern Emerg Med. 2022; 17(8):2199-2208). Also, in a German study, the CFQ-11 of 42 patients followed with severe fatigue after approximately one year was 21±5 (Kedor C. et al. Nat Commun. 2022;13(1):5104), and values varied among the patients studied. Considering the patient population used in previous sample size calculations, the CFQ of 15 ± 5 at several months after onset and the CFQ-11 of 16.7 ± 5.6 after an average of 430 days of follow-up in Ireland seem consistent. Therefore, we assumed a CFQ of 15 ± 5 at one year after onset and set the minimal important difference to be a 3-point drop in the CFQ-11 score. Assuming a dropout rate of about 25%, we calculated α error = 0.5 and β error = 0.20, resulting in a total of 120 patients (60 in each group), unchanged from the sample size required for the original analysis.

9 Policies on obtaining consent, clinical trial records, data protection, and publication; clinical trial management organization

9.1 Approval of clinical trials, information to patients and obtaining consent

This clinical trial will be initiated only after the Investigational Review Board (IRB) has reviewed and approved all required legal documents in accordance with GCP standards. This applies equally to conduct after the regulations have been amended.

Obtain consent from each patient prior to participation in the clinical trial. Consent should be obtained in writing (paper) or by electronic signature. When consent is obtained in paper form, a copy of the explanation document and signed consent form is provided to the patient. If consent is obtained electronically, it is performed remotely using eConsent, and the written explanation and electronically signed consent form are provided to the patient.

The investigator must provide sufficient explanation to the patient using the explanatory document. Language that can be understood by the patient should be used, and technical terms and expressions should be avoided to the greatest extent possible.

The investigator must also give the patient sufficient time to consider participation in the study, and obtain the patient's free and voluntary consent after confirming that the patient understands the content of the consent form. The investigator signs the consent form (including electronic signature) in the same manner as the patient. If a collaborator provides supplementary explanations, the collaborator must also sign the consent form (including electronic signature).

Re-consent must be obtained in the event of new significant findings regarding this clinical trial.

9.2 Data Quality Assurance

Data on all subjects in this clinical trial will be recorded on paper or electronic case report forms. The investigator is

responsible for verifying that the entries are accurate and correct by electronically signing the electronic case report form. The investigator (sub)investigator should keep accurate records (source documents) to support the information provided in the electronic case report form.
Monitoring personnel shall conduct ongoing source document review to ensure that electronic case report data entered by authorized site personnel are accurate and verifiable from source documents, that the safety and rights of subjects are protected, and that the study is conducted in accordance with the study protocol (and other study-related documents) and all applicable regulatory requirements, The investigator confirms that the data in the electronic case report is accurate and verifiable from source documents, that the safety and rights of subjects are protected, and that the study is being conducted in accordance with the protocol (and other clinical trial documents) and all applicable regulatory requirements.

9.3 record

9.3.1 original source

All source documents shallbe accurate, clear, unambiguous, unaltered, and available forinspection. The investigator (sub)investigator is responsible for ensuring that the accuracy, legibility, contemporaneity, originality, and pneumaticity of source documents are maintained, regardless of whether the data are written on paper or entered electronically. Original documents should not be obscured by the use of correction fluid or temporarily attached to documents (e.g., by the use of sticky notes). Copies of case report forms, etc. shall not be considered source documents.
Source documents are the original records from which raw data were first recorded. Source documents include hospital/clinic/general practitioner records, medical records, laboratory results, printed materials, pharmacy records, medical records, completed rating scales, etc. Original documents are stored in a secure location with restricted access.
Source documents created by computer and stored electronically shall be printed for review by the monitoring staff if they are not accessible from the medical record, etc. and cannot be reviewed by the monitoring staff. The printed materials shall be signed and dated by the investigator(s) responsible for the study and kept as the source documents for the subject concerned. The source data shall also comply with all laws and regulations regarding the use of electronic records and electronic signatures via computer systems or other electronic systems.

9.3.2 Direct access to original documents

The director of the investigational site and the investigator(s) should permit monitoring, audits, investigations by the investigational review committee, and inspections by regulatory authorities related to the clinical trial, and should allow direct access to the source documents. In the direct inspection, sufficient attention must be paid to the protection of the privacy of subjects.

To confirm that the clinical trial is being conducted appropriately and that the reliability of data is sufficiently ensured, the person in charge of auditing will conduct audits and directly inspect source documents and other clinical trial-related records at the site.

The monitors will monitor the conduct of the clinical trial using a combination of central monitoring, on-site monitoring and off-site monitoring. The investigator (or coordinator) will discuss the identification of source documents and the

method of direct inspection with the investigator in advance.

9.3.3 Retention period for records

9.3.3.1 Clinical Trial Review Committee

The establisher of a clinical trial review committee shall retain the Standard Operating Procedures, roster of committee members, submitted documents, records and summaries of meetings, letters and other records until the later date of either 1) or 2) below. However, if the coordinating investigator or the principal investigator requires a longer retention period, the investigator shall discuss the retention period and method with the coordinating investigator or the principal investigator.

1) The date of post-marketing approval for the investigational drug (or three years after the date of decision to discontinue development, if development has been discontinued)
2) Date 3 years have elapsed since the discontinuation or termination of the clinical trial

9.3.3.2 Implementing medical institution

The head of the site shall retain the documents or records pertaining to the clinical trial that should be retained at the site until the later of 1) or 2) below. However, if the coordinating investigator or the principal investigator requires a longer retention period, the period and method of retention shall be discussed with the coordinating investigator or the principal investigator. (2) A person responsible for the retention of each record shall be appointed for each record and the records shall be retained.

The head of the implementing medical institution or the person responsible for keeping the records will take steps to ensure that these records are not lost or destroyed during this retention period and that they are available upon request.

1) The date of post-marketing approval for the investigational drug (or three years after the date of decision to discontinue development, if development has been discontinued)
2) Date 3 years have elapsed since the discontinuation or termination of the clinical trial

9.3.3.3 Persons who conduct clinical trials on their own

(2) The person conducting the clinical trial shall preserve documents and records related to the clinical trial as stipulated in GCP. The retention period of documents and records shall be until the later of the following dates.

These documents should be kept in a safe place and stored so that they can be retrieved quickly and easily when needed, such as for audits or inspections.

1) The date of post-marketing approval for the investigational drug (or three years after the date of decision to discontinue development, if development has been discontinued)
2) Date 3 years have elapsed since the discontinuation or termination of the clinical trial

9.4 Emergency Reporting of Adverse Events

The coordinating investigator is responsible for reporting to the regulatory authorities in accordance with the "Procedures

for Handling Safety Information".

9.5 Confidentiality and patient privacy

Measures for data protection and data confidentiality shall be implemented for the collection, storage and processing of patient data. Each patient data obtained in this clinical trial is confidential information and is forbidden to be disclosed to third parties with the following exceptions

The patient's attending physician or other health care professional responsible for the patient's well-being shall have access to the treatment data.

Access to data obtained at the site as a result of the clinical trial with respect to inspections as required by the investigator, IRB and regulatory authorities.

9.6 Clinical Trial Management Organization

9.6.1 Clinical Trial Coordinating Physician

Kensuke Nakamura

Associate Professor, Department of Emergency Medicine, Teikyo University School of Medicine, Department of Emergency Medicine, Center for Advanced Emergency Medical Care, Hospital

Address: 2-11-1 Kaga, Itabashi-ku, Tokyo 173-8606

TEL: 03-3964-1211

9.6.2 medical specialist

In this clinical trial, it will be established for the purpose of providing advice on the conduct and management of the clinical trial.

Shigeki Fujitani

Professor of Emergency Medicine, St. Marianna University School of Medicine

Address: 2-16-1 Sugo, Miyamae-ku, Kawasaki, Kanagawa, 216-8511, Japan

TEL: 044-977-8111

9.7.3 Development of Therapeutic Biomarkers

Kazuhiro Kondo

Professor, The Jikei University School of Medicine

Address: 3-25-8 Nishi-Shinbashi, Minato-ku, Tokyo 105-8461

tel: 03-3433-1111

9.7.4 Clinical trial site

The details are described in the Appendix "Implementation Structure".

9.7.5 Investigational drug providers

N/A.

9.7.6 Monitoring

The details are described in the Appendix "Implementation Structure".

9.7.7 Auditing

The details are described in the Appendix "Implementation Structure".

9.7.8 Registration Office

The details are described in the Appendix "Implementation Structure".

9.7.9 Statistical Analysis

Masahiro Goto
TXP Medical Corporation
Address: Entrepreneur Lab 252, University of Tokyo, 7-3-1 Hongo, Bunkyo-ku, Tokyo 113-8485, Japan
tel: 03-5615-8433

10 References

10.1 public documents

*1: Chalder, *T.; Berelowitz, G.; Pawlikowska, T.; Watts, L.; Wessely, S.; Wright, D.; Wallace, E. P. (1993). "Development of a fatigue scale". Journal of Psychosomatic Research.* ***37*** *(2): 147-153. ISSN 0022-3999. PMID 8463991.*

10.2 unpublished data

None
