# Supplementary material for: Donepezil for Fatigue and Psychological Symptoms in Post–COVID-19 Condition: A Randomized Clinical Trial
Source: JAMA Netw Open. 2025 Mar 17;8(3):e250728. doi: 10.1001/jamanetworkopen.2025.0728 (PMC11915061; doi:10.1001/jamanetworkopen.2025.0728)
Supplement: Supplement 2. — eTable 1. Medical History and Comorbidities of Participants eTable 2. Number of Missing Data in the Full Analysis Set eTable 3. Characteristics of the Patients Lost to Follow-Up eTable 4. An Absolute Value of Each Outcome in the Full Analysis Set eTable 5. An Absolute Value of Each Outcome in the Per-Protocol Set eTable 6. Subgroup Analyses Based on Age, Sex, Medical History, and Disease Severity [file jamanetwopen-e250728-s002.pdf]

Supplementary Online Content

Nakamura K, Kondo K, Oka N, et al. Donepezil for fatigue and psychological symptoms in post–COVID-19 condition: a randomized clinical trial. *JAMA Netw Open.* 2025;8(3):e250728. doi:10.1001/jamanetworkopen.2025.0728

eTable 1. Medical History and Comorbidities of Participants
eTable 2. Number of Missing Data in the Full Analysis Set
eTable 3. Characteristics of the Patients Lost to Follow-Up
eTable 4. An Absolute Value of Each Outcome in the Full Analysis Set
eTable 5. An Absolute Value of Each Outcome in the Per-Protocol Set
eTable 6. Subgroup Analyses Based on Age, Sex, Medical History, and Disease Severity

This supplementary material has been provided by the authors to give readers additional information about their work.

© 2025 Nakamura K et al. *JAMA Network Open*.

eTable 1. Medical History and Comorbidities of Participants

Group	Donepezil	Control
n	55	55
Pollen allergy	17	13
Zinc deficiency	15	12
Migraine	7	6
Insomnia	9	2
Dyslipidemia	4	7
Constipation	4	6
Hypertension	2	7
House dust allergy	4	3
Reflux esophagitis	1	6
Allergic rhinitis	0	6
Iron deficiency anemia	4	2
Dry eye	2	3
Lipase increase	3	2
Premenstrual syndrome	4	1
Bronchial asthma	2	3
Adaptation disorder	3	2
Copper increase	3	2
Liver dysfunction	2	2
Lumbar pain	2	2
Hyperuricemia	2	2

© 2025 Nakamura K et al. *JAMA Network Open*.

eTable 2. Number of Missing Data in the Full Analysis Set

Group	Donepezil	Control
N	55	55
Age, year	0	0
Sex, female	0	0
Concomitant therapy	0	0
Symptoms on inclusion	0	0
Number of vaccinations	0	0
Body temperature, °C	0	0
Systolic pressure, mmHg	0	0
Diastolic pressure, mmHg	0	0
Heart rate, /min	0	0
Respiratory rate, /min	1	0
Oxygen saturation, %	0	0
White blood cells, 10^9/L	1	1
Neutrophils, %	1	1
Lymphocytes, %	2	1
Hemoglobin, g/dL	2	1
Platelets, 10^9/L	1	1
Albumin, g/dL	1	0
AST, U/L	1	0
ALT, U/L	1	0
LDH, U/L	1	0
BUN, mg/dL	1	0
Creatinine, mg/dL	1	0
Hemoglobin A1c, %	1	0
CRP, mg/dL	6	6
CFS		
Pre-intervention	0	0
3 weeks after the intervention	0	0
8 weeks after the intervention	1	2
HADS		
Pre-intervention	0	0
3 weeks after the intervention	0	0
8 weeks after the intervention	0	0
IES-R		
Pre-intervention	0	0
3 weeks after the intervention	0	0
8 weeks after the intervention	0	0
EQ-5D-5L		
Pre-intervention	0	0
3 weeks after the intervention	0	0
8 weeks after the intervention	1	2
PHQ-9		
Pre-intervention	0	0
3 weeks after the intervention	0	0
8 weeks after the intervention	0	0
Daily health status		
Pre-intervention	0	0
3 weeks after the intervention	0	0
8 weeks after the intervention	1	2

AST, aspartate aminotransferase; ALT, alanine aminotransferase; LDH, lactate dehydrogenase; BUN, blood urea nitrogen; CRP, C-reactive protein; CFS, Chalder Fatigue Scale; HADS, Hospital Anxiety and Depression Scale; IES-R, Impact of Event Scale-Revised; EQ-5D-5L, EuroQol-5Dimension-5Level; PHQ-9, Patient Health Questionnaire

© 2025 Nakamura K et al. *JAMA Network Open*.

eTable 3. Characteristics of the Patients Lost to Follow-Up

Group	Donepezil	Control
N	5	5
Age, year	44 (10)	36 (9)
Sex, male	1 (20%)	2 (40%)
Sex, female	4 (80%)	3 (60%)
Symptoms on inclusion		
Cough	2 (40%)	1 (20%)
Sputum	1 (20%)	2 (40%)
Fever	1 (20%)	0 (0%)
Taste disorder	0 (0%)	2 (40%)
Appetite loss	2 (40%)	1 (20%)
Number of vaccinations		
0	0 (0%)	2 (40%)
1	0 (0%)	0 (0%)
2	1 (20%)	1 (20%)
3	3 (60%)	1 (20%)
4	0 (0%)	1 (20%)
5	1 (20%)	0 (0%)
6	0 (0%)	0 (0%)
Vital signs on inclusion		
Body temperature, °C	36.7 (0.1)	36.6 (0.5)
Systolic pressure, mmHg	117 (19)	123 (20)
Diastolic pressure, mmHg	73 (8)	76 (14)
Heart rate, /min	76 (8)	72 (8)
Respiratory rate, /min	17 (5)	14 (2)
Oxygen saturation, %	97 (1)	97 (1)
Laboratory findings on inclusion		
White blood cells, 10^9/L	6.8 (1.1)	5.8 (1.0)
Neutrophils, %	62 (6)	56 (8)
Lymphocytes, %	31.6 (7.1)	33.5 (6.8)
Hemoglobin, g/dL	13.9 (1.3)	14.0 (1.7)
Platelets, 10^9/L	286 (13)	303 (63)
Albumin, g/dL	4.5 (0.5)	4.6 (0.2)
AST, U/L	17.3 (2.9)	21.8 (7.9)
ALT, U/L	17 (5)	27 (20)
LDH, U/L	143 (24)	153 (29)
BUN, mg/dL	11.7 (1.5)	12.6 (3.3)
Creatinine, mg/dL	0.65 (0.08)	0.64 (0.15)
Hemoglobin A1c, %	5.28 (0.22)	5.38 (0.16)
CRP, mg/dL	0.048 (0.021)	0.075 (0.037)

CPAP, continuous positive airway pressure; AST, aspartate aminotransferase; ALT, alanine aminotransferase; LDH, lactate dehydrogenase; BUN, blood urea nitrogen; CRP, C-reactive protein

© 2025 Nakamura K et al. *JAMA Network Open*.

eTable 4. An Absolute Value of Each Outcome in the Full Analysis Set

Group<br>n	Donepezil<br>55	Control<br>55	p<br>value
CFS			
Pre-intervention	22.3 ± 4.5	21.4 ± 4.4	0.31
3 weeks after the intervention	14.6 ± 6.9	14.1 ± 6.7	0.67
8 weeks after the intervention	12.6 ± 6.0	13.4 ± 6.4	0.52
HADS			
Pre-intervention	14.0 ± 7.2	13.7 ± 6.8	0.84
3 weeks after the intervention	10.7 ± 7.3	11.8 ± 7.2	0.42
8 weeks after the intervention	10.6 ± 7.7	10.5 ± 8.0	0.94
IES-R			
Pre-intervention	53.5 ± 16.0	49.5 ± 15.3	0.18
3 weeks after the intervention	43.8 ± 17.4	41.6 ± 14.5	0.47
8 weeks after the intervention	41.7 ± 18.4	39.4 ± 16.9	0.49
EQ-5D-5L			
Pre-intervention	10.6 ± 3.7	10.0 ± 3.1	0.37
3 weeks after the intervention	8.8 ± 3.3	8.4 ± 3.3	0.52
8 weeks after the intervention	8.3 ± 3.3	8.2 ± 3.2	0.96
PHQ-9			
Pre-intervention	20.6 ± 5.3	21.2 ± 5.3	0.60
3 weeks after the intervention	17.7 ± 5.8	17.8 ± 5.0	0.92
8 weeks after the intervention	16.6 ± 5.7	16.1 ± 5.6	0.65
Daily health status			
Pre-intervention	49.2 ± 20.0	53.3 ± 18.1	0.26
3 weeks after the intervention	56.3 ± 20.8	60.0 ± 17.9	0.31
8 weeks after the intervention	60.4 ± 23.6	60.8 ± 18.9	0.90

CFS, Chalder Fatigue Scale; HADS, Hospital Anxiety and Depression Scale; IES-R, Impact of Event Scale-Revised; EQ-5D-5L, EuroQol-5Dimension-5Level; PHQ-9, Patient Health Questionnaire

© 2025 Nakamura K et al. *JAMA Network Open*.

eTable 5. An Absolute Value of Each Outcome in the Per-Protocol Set

Group<br>n	Donepezil<br>54	Control<br>52	p<br>value
CFS			
Pre-intervention	22.3 ± 4.6	21.3 ± 4.4	0.27
3 weeks after the intervention	14.7 ± 7.0	14.0 ± 6.7	0.59
8 weeks after the intervention	12.6 ± 6.0	13.4 ± 6.3	0.53
HADS			
Pre-intervention	13.9 ± 7.2	13.7 ± 6.9	0.84
3 weeks after the intervention	10.8 ± 7.4	11.9 ± 7.3	0.43
8 weeks after the intervention	10.7 ± 7.8	10.3 ± 8.1	0.82
IES-R			
Pre-intervention	53.4 ± 16.1	49.4 ± 15.4	0.19
3 weeks after intervention	44.0 ± 17.5	41.5 ± 14.7	0.43
8 weeks after intervention	41.9 ± 18.5	39.6 ± 17.4	0.50
EQ-5D-5L			
Pre-intervention	10.6 ± 3.7	10.0 ± 3.1	0.38
3 weeks after the intervention	8.9 ± 3.3	8.4 ± 3.3	0.47
8 weeks after the intervention	8.3 ± 3.3	8.3 ± 3.3	0.90
PHQ-9			
Pre-intervention	20.6 ± 5.4	21.0 ± 5.3	0.68
3 weeks after the intervention	17.8 ± 5.8	17.8 ± 5.1	0.98
8 weeks after the intervention	16.7 ± 5.8	15.9 ± 5.6	0.49
Daily health status			
Pre-intervention	49.0 ± 20.1	54.0 ± 18.0	0.18
3 weeks after the intervention	56.2 ± 21.0	59.8 ± 18.2	0.35
8 weeks after the intervention	60.1 ± 23.7	61.0 ± 19.1	0.83

CFS, Chalder Fatigue Scale; HADS, Hospital Anxiety and Depression Scale; IES-R, Impact of Event Scale-Revised; EQ-5D-5L, EuroQol-5Dimension-5Level; PHQ-9, Patient Health Questionnaire

© 2025 Nakamura K et al. *JAMA Network Open*.

eTable 6. Subgroup Analyses Based on Age, Sex, Medical History, and Disease Severity

	Estimating treatment effect using regression model adjusting for baseline CFS mean difference (95%CI)	P Value
Age		
18-39	0.340 (-3.362, 4.043)	0.854
≥40	0.219 (-3.472, 3.910)	0.906
Sex		
Male	0.386 (-3.512, 4.284)	0.843
Female	0.415 (-3.193, 4.024)	0.819
Comorbidity		
Mental diseases		
Yes	2.360 (-4.55, 9.244)	0.478
No	-0.035 (-2.861, 2.792)	0.981
Digestive diseases		
Yes	-1.854 (-6.833, 3.125)	0.452
No	1.148 (-1.962, 4.257)	0.465
Endocrine diseases		
Yes	-0.171 (-4.538, 4.197)	0.937
No	0.861 (-2.366, 4.088)	0.596
Allergy		
Yes	-0.112 (-4.833, 4.609)	0.962
No	0.598 (-2.541, 3.737)	0.705
Neurological diseases		
Yes	-4.141 (-12.594, 4.312)	0.316
No	1.256 (-1.522, 4.035)	0.371
Respiratory diseases		
Yes	-3.648 (-13.032, 5.737)	0.416
No	0.589 (-2.118, 3.295)	0.667
Cardiovascular diseases		
Yes	-1.260 (-14.192, 11.671)	0.824
No	-0.110 (-2.848, 2.629)	0.937
Severity		
Low	1.066 (-1.722, 3.854)	0.449
Moderate-high	-3.733 (-11.548, 4.082)	0.323

CFS, Chalder Fatigue Scale; CI, confidence interval

© 2025 Nakamura K et al. *JAMA Network Open*.
