# Supplementary material for: Donepezil for Fatigue and Psychological Symptoms in Post–COVID-19 Condition: A Randomized Clinical Trial
Source: JAMA Netw Open. 2025 Mar 17;8(3):e250728. doi: 10.1001/jamanetworkopen.2025.0728 (PMC11915061; doi:10.1001/jamanetworkopen.2025.0728)
Supplement: Supplement 3. — Data Sharing Statement [file jamanetwopen-e250728-s003.pdf]

Data Sharing Statement

Nakamura. Donepezil for Fatigue and Psychological Symptoms in Post–COVID-19 Condition. *JAMA Netw Open*. Published March 17, 2025. doi:10.1001/jamanetworkopen.2025.0728

Data
Additional Information: The Japan Registry of Clinical Trials Identifier: jRCT 2031220510
Data available: Yes
Data types: Other (please specify)
Additional Information: The datasets generated and analyzed during the present study are available from the corresponding author upon reasonable request.
How to access data: The datasets generated and analyzed during the present study are available from the corresponding author upon reasonable request.
When available: With publication

Supporting Documents
Document types: None

Additional Information
Who can access the data: The datasets generated and analyzed during the present study are available from the corresponding author upon reasonable request.
Types of analyses: for any purpose or for a specified purpose
Mechanisms of data availability: with investigator support
